# ReRep: Computational detection of repetitive sequences in genome survey sequences (GSS)

**DOI:** 10.1186/1471-2105-9-366

**Published:** 2008-09-09

**Authors:** Thomas D Otto, Leonardo HF Gomes, Marcelo Alves-Ferreira, Antonio B de Miranda, Wim M Degrave

**Affiliations:** 1 Laboratory for Functional Genomics and Bioinformatics, IOC, Fiocruz, Rio de Janeiro, Brazil; 2 Fundação Ataulpho de Paiva, Rio de Janeiro, Brazil; 3 Medicine Faculty, UFRJ, Rio de Janeiro, Brazil

## Abstract

**Background:**

Genome survey sequences (GSS) offer a preliminary global view of a genome since, unlike ESTs, they cover coding as well as non-coding DNA and include repetitive regions of the genome. A more precise estimation of the nature, quantity and variability of repetitive sequences very early in a genome sequencing project is of considerable importance, as such data strongly influence the estimation of genome coverage, library quality and progress in scaffold construction. Also, the elimination of repetitive sequences from the initial assembly process is important to avoid errors and unnecessary complexity. Repetitive sequences are also of interest in a variety of other studies, for instance as molecular markers.

**Results:**

We designed and implemented a straightforward pipeline called ReRep, which combines bioinformatics tools for identifying repetitive structures in a GSS dataset. In a case study, we first applied the pipeline to a set of 970 GSSs, sequenced in our laboratory from the human pathogen *Leishmania braziliensis*, the causative agent of leishmaniosis, an important public health problem in Brazil. We also verified the applicability of ReRep to new sequencing technologies using a set of 454-reads of an *Escheria coli*. The behaviour of several parameters in the algorithm is evaluated and suggestions are made for tuning of the analysis.

**Conclusion:**

The ReRep approach for identification of repetitive elements in GSS datasets proved to be straightforward and efficient. Several potential repetitive sequences were found in a *L. braziliensis *GSS dataset generated in our laboratory, and further validated by the analysis of a more complete genomic dataset from the EMBL and Sanger Centre databases. ReRep also identified most of the *E. coli *K12 repeats prior to assembly in an example dataset obtained by automated sequencing using 454 technology. The parameters controlling the algorithm behaved consistently and may be tuned to the properties of the dataset, in particular to the length of sequencing reads and the genome coverage. ReRep is freely available for academic use at .

## Background

Repetitive sequences make up a significant part of many genomes [1]. They are dynamic elements that contribute to plasticity, and they generally evolve faster than coding regions; for this reason they can be used in species identification and phylogenetic inference [2]. Current genome sequencing methodologies involve mostly high-throughput shotgun approaches, and Genome Survey Sequencing (GSS) is often an initial but large-scale step. The presence of a considerable amount of repetitive sequences in the genome under study can hamper library construction, accurate sequencing and especially assembly of the final genome sequence. An early assessment of the nature, frequency and variability of the repetitive content of a genome is therefore important. Such data strongly influence the evaluation of sequencing strategies and assembly because of the impact of repetitive elements on the estimation of genome coverage, library quality and progress in scaffold construction. The elimination of repetitive sequences from the initial assembly process is important to avoid errors and unnecessary complexity. Identified repeats can also be useful in a variety of other studies, for instance as molecular markers in mapping and strain characterization. For example, prior to large-scale sequencing of the *Leishmania braziliensis *genome, most of its known repeats were simple microsatellites or species-specific repeats [3-5], while for *Leishmania major*, a closely related species, several other types of repeats were reported [6]. A global analysis of the types and frequencies of repeats of *L. major *could only be done after completion of the sequence [7].

Algorithms for *de novo *repeat detection [8] are normally based on suffix trees [9,10], on word count algorithms [11,12] or on similarity searches [13]. Programs like Repeatmasker (Smit, unpublished) search for repeats using a database of known repetitive sequences, such as Repbase [14]. However, the high evolutionary rates make detection of a particular repeat feasible only in closely related organisms, and many repeats are species-specific [1]. It is important to note that these programs usually require a complete and accurate genome assembly while, on the other hand, the presence of repeat sequences greatly hampers the assembly process in many eukaryotes, such as in *Trypanosoma cruzi *[15], human [16] or Drosophila [17]. One of the difficulties in repeat recognition in GSS data arises when repeats are longer than the reads. During the assembly process these repeats tend to be joined into one contig [18], but ideally, reads with repetitive sequences should be excluded from the initial assembly and mapped manually in the final stages [18]. Identifying repetitive units before the assembly thus avoid errors and speeds up the process, providing more accurate scaffolds. However, it is difficult to detect and estimate the frequency of repeats when working with a small dataset (e.g. at low genome coverage), and it is hard to differentiate between a truly repetitive sequence and a genomic region with higher sequencing coverage.

To help identify repetitive units before the assembly phase of a genome, we designed and evaluated a pipeline (ReRep – Read Repeat Finder) based on similarity searches [19,20], the interpretation of sequence landscapes [21], the assembly of clustered sequences [22] and in-house Perl scripts. The main challenge is to determine the limits of the repetitive sequences found in the GSS dataset and to estimate their abundance in the whole genome.

As a case study, we used 970 GSS with at least 150 bp of good quality (Phred quality Q >= 20 [23]) generated in our laboratory and covering approximately 1.4% of the genome of *L. braziliensis*. Several putative repetitive structures could be identified with our approach. Results were then verified against several datasets representing about 16% coverage, such as *L. braziliensis *GSS obtained from EMBL (described in [24]), from the Sanger Centre (Whole Genome Shotgun sequences, WGS) and against the complete assembled genome. Human leishmaniasis, a tropical disease transmitted by phlebotomine sand flies, is caused by protozoan parasites of the genus *Leishmania*. *L. braziliensis *is the most common etiological agent of cutaneous leishmaniasis in Brazil and the disease constitutes an important public health problem in Brazil and other Central and South American countries [25]. We also applied ReRep to a large set of reads obtained from a genome project of *E. coli *K12 using 454 technology, to ascertain the viability of the methodology with the shorter reads from this approach. We could also determine the number of false positives and negatives in this experiment, as the genome is completely assembled.

## Results

### Design and testing of ReRep

**Figure 1:**
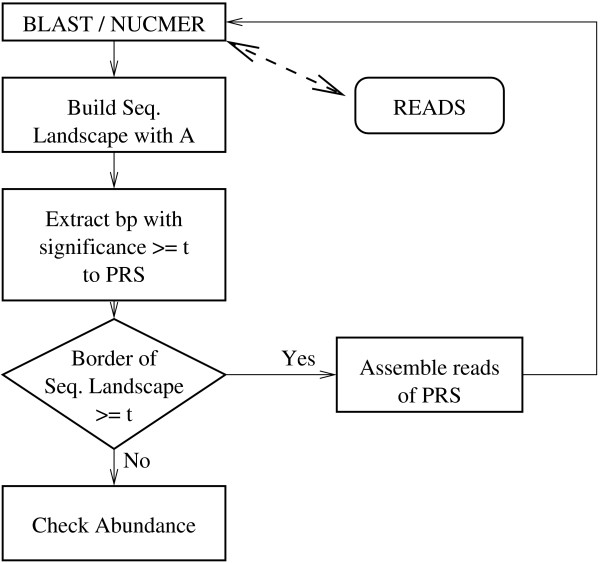
**Pipeline of ReRep for detection of repetitive elements**. GSS sequences are cross-compared (all-against-all) using BLAST or NUCMER. For each read, all alignments of minimal length *l *enter into the construction of its sequencing landscape. If sub-sequences of a sequence landscape occur with a frequency higher than the chosen threshold *t*, they are considered to be putative repetitive sequences (PRS). PRSs are extended if the base frequencies of at least one border of the sequence landscape are above the threshold *t*, by assembling all sequences included in the construction of its sequence landscape. If more GSSs are provided, the cycle is repeated.

**Figure 2:**
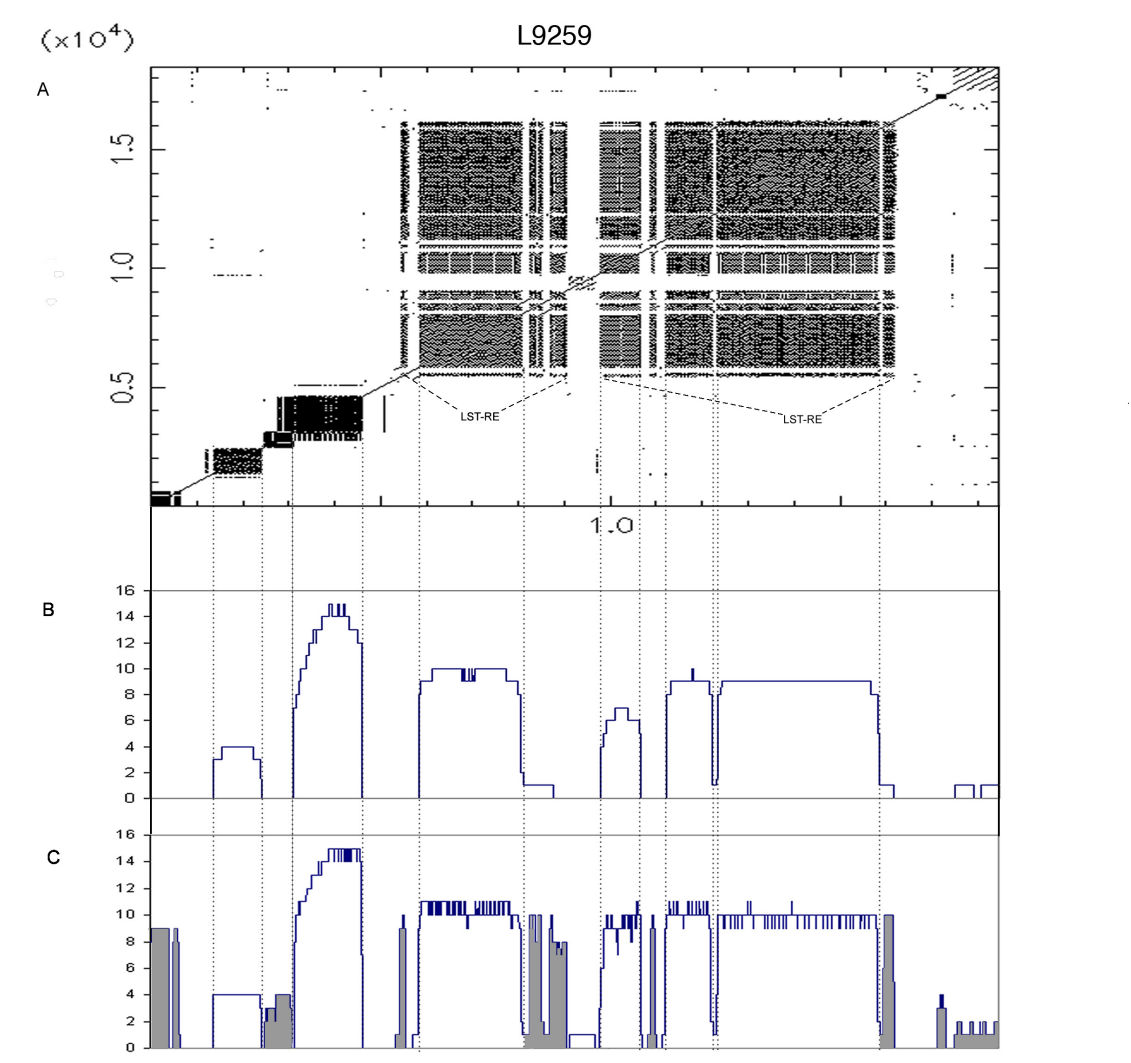
**Representation of cosmid L9259 with sequence landscapes**. The vertical dotted lines in the graph indicate the borders of putative repetitive elements. (A) Dotplot (word size 8) of cosmid L9259 against itself. If 8 bases are equal, a point is set. Repetitive regions are clearly visible. Regions containing copies of LST-RE [3], a 91 base pattern repeated in tandem, are indicated. (B) Sequence landscape of cosmid L9259 (*l *= 400). The x axis represents the position of each residue in the cosmid. It correlates directly with the position in the dotplot. The y axis plots the occurrence of each residue of the cosmid (the residue positions contained in a High-scoring Segment Pair) included in alignments with a minimal length of 400 bp. (C) As in B, with *l *= 50. Shorter repetitive elements are now visible, marked in grey.

During GSS sequencing in a larger project, and usually before assembly, repetitive elements should be identified and temporarily removed from the dataset. For this purpose, we developed a workflow (Figure 1) where GSS sequences are cross-compared (all-against-all) using BLAST [19] or NUCMER from the MUMmer package [20]. To speed up the algorithm, the first seed (word size with default value of 11) can be increased. For each read, all alignments of minimal length *l *enter into the construction of the sequencing landscape, a graphical representation of the abundance of each base in the sequence (Figure 2B). If sub-sequences of a landscape occur with a frequency higher than the chosen threshold *t*, they are considered to be Putative Repetitive Sequences (PRS). PRSs are extended if the base frequencies of at least one border of the sequence landscape are above the threshold *t*, by assembling all sequences included in the construction of its sequence landscape. The cycle is repeated to find shorter or less abundant repetitions, as shown in Figure 1.

To test our ReRep pipeline, we first applied it to cosmid L9259 of *L. major*, which had previously been analyzed for the presence of repeats [6]. Figure 2 shows a dotplot (word size 8) of cosmid L9259 against itself, showing the repetitive structures present in this sequence. Also shown are two corresponding Sequence Landscapes (SLs – Figure 2B and 2C). The SL represents the frequenc y of each base as a participant in a High Scoring Pair (HSP), generated from alignments with length bigger than *l*. In this case for the value of *l *we chose 400 and 50. In general, one is interested initially in finding the larger repeats. As a rule of thumb, one can start with an *l *value of half the size of the average read length. The user can define the sensitivity of repeat detection by adjusting the parameter *t *(threshold). In fully assembled genomes *t *should be one, while for GSS analysis, *t *must be adapted to the degree of coverage. Even with low coverage, simple overlaps of sequences can be interpreted as repeats, thus *t *must be sufficiently high to minimize the number of false positives.

### Analysis of *L. braziliensis *GSS datasets

Analysis of an experimental dataset containing 970 *L. braziliensis *GSS reads generated in our laboratory allowed the detection of five Putative Repetitive Sequences PRS (named PRS_1 – 5) using the parameters set *l *= 400, *t *= 2. These PRSs and the respective SLs can be seen in the additional file 1: PRS. PRS_1 is composed of several *in tandem *repetitions of a shorter element, called TanPRS_1, visualized after analysis of PRS_1 with the EMBOSS program "Etandem" [26]. Sequencing of a PCR product with primers designed to amplify the PRS_1 element (Primers: forward TTGTAAAACGACGGCCAGTG, reverse CACACAGGAAACAGCTATGAC) confirmed the existence and structure of the tandem repeat. The size of the largest fragment obtained by PCR was around 2 kb (See additional file 2: PCR). As the larger element PRS_1 contains such tandem repeats, its copy number cannot be found precisely by the analysis of BLAST [19] results, nor by algorithms based on word counting; the analysis of the shorter repetitive elements (TanPRS_1) gives a better estimate of their frequency. The copy number of TanPRS_1 in our 970_GSS dataset, using different mismatch scores, was equal to 9, 19, 20 and 20 when the allowed percentage of mismatches was set to 0%, 5%, 10% and 15%, respectively. For comparison, we assembled a dataset containing 9644 *L. braziliensis *GSS from the EMBL database; a dataset of the first 9693 WGS reads from the *L. braziliensis *genome project at the Sanger Centre and a set of 9644 simulated reads generated from the *L. braziliensis *assembled genome sequence (Sanger Centre) using the program 'ReadSimulator' (Huson, unpublished). Finally, the complete assembled genome was used to obtain the best possible copy numbers. As the final assembly of the genome of *L. braziliensis *contains a high number of gaps, the number of repeats could be wrong. Under the same conditions mentioned above, we obtained for TanPRS_1 in the 9644_GSS_EMBL frequencies of 67, 136, 145, 145 and compared to true copy numbers of 506, 933, 940, 941 in the assembled genome. A multiple alignment shows few mismatches between the TanPRS_1 elements (see additional files 3 and 4: Align_PRS_1 and Align_TanPRS_1). PRS_2 was found twice in the 970_GSS dataset, and 5 times in the complete genome, while PRS_3 was identified by ReRep as a PRS but, after verification against the complete genome sequence, was shown to be present only once (false positive); a subregion of this PRS (97 bp) has similarity to a single copy DNA in *L. major *and *L. infantum*. PRS_4 was found 4 times in the 970_GSS dataset, and 42 times in the genome (considering the results obtained with an e-value of e^-20^). PRS_5 was found 4 times in 970_GSS, 29 times in 9644_GSS_EMBL and 79 times in the complete genome, and was identified as a subtelomeric region of *L. braziliensis *[4], occurring in 24 chromosomes. PRS_5 was also found in *L. peruviana*, *L. panamensis *and *L. guyanensis*, but neither in *L. major *nor in *L. infantum*.

Additional analyses with Repeatmasker and Repbase [14] revealed no known repeats in 970_GSS, including repetitive elements previously described by other groups for the genus *Leishmania *([4,7]). Thus, less than 0.93% of the 970_GSS dataset is composed of simple repeats and low-complexity regions. Table 1 shows the length of each PRS, the number of reads and chromosomes containing the PRS and the frequency of each repeat in the respective datasets.

**Table 1:** Number of occurrence of putative repetitive sequences (PRS) in datasets

Name	Length	A	B	C	# reads in A	# reads in B	# reads in C
PRS_1	717	4	67	885	2	16	2
PRS_2	452	2	4	5	2	4	1
PRS_3	541	2	2	1	2	2	1
PRS_4	729	4	5	42	3	4	2
PRS_5	566	4	29	79	4	29	24
TanPRS_1	61	21	138	933	2	16	2

The number of occurrence of each PRS was calculated after parsing BLAST results using an e-value with cut-off e^-20 ^in the following datasets: (A) 970_GSS; (B) 9644_GSS_EMBL; and (C) the complete assembled genome of *L. braziliensis*. Number of reads (#reads) in dataset A and B and chromosomes (#Chr.) in dataset C where the corresponding PRS was found.

### Minimizing false positives by adjusting the parameter *t*

In order to minimize the number of false positives detected by ReRep, we analyzed the behaviour of the *t *parameter, which is related to the sequencing coverage and the abundance of the PRSs. We applied ReRep to the three reference datasets 9644_GSS_EMBL, 9693_WGS_Sanger and 9644_simGSS_Sanger with parameters *l *= 400 and *t *from 1 to 5; we also used *t *= 6 for the 9644_GSS_EMBL dataset. The results of the 16 runs were analyzed against the whole genome sequence obtained by BLAST to check the abundances of the PRSs, broken down by the number of hits per PRS (Table 2).

**Table 2:** Detection of PRS with different *t *parameter in the three reference datasets

	9644_GSS_EMBL						9693_WGS_Sanger					9644_simGSS_Sanger				
t	1	2	3	4	5	6	1	2	3	4	5	1	2	3	4	5

# PRS	###	330	121	39	21	10	500	57	21	12	10	415	30	18	10	7

# no hits	42	19	7	4	3	1	10	1	0	0	0	0	0	0	0	0
# 1 hit	###	225	78	21	12	5	366	25	2	0	0	300	5	1	0	0
# 2 hits	94	33	7	2	0	0	40	6	1	0	0	34	0	0	0	0
#>2 hits	128	53	29	12	6	4	84	25	18	12	10	81	25	17	10	7
#>30 hits	19	11	9	5	3	3	13	8	8	8	8	19	14	11	9	7

The number of PRSs found by ReRep with different *t *parameter for the three datasets is given in the first line (#PRS). The detected PRSs of each individual run are BLASTed against the whole genome sequence (e-value e^-20^). The number of hits is reported in the following lines: # no hit and # 1 hit indicates false positives. # > 2 hits indicate PRSs occurring more than twice in the genome. # > 30 hits indicates highly repetitive PRSs. An *l *parameter of 400 was used.

The value of *t *is directly correlated with specificity (and false negatives), and inversely with sensitivity (and false positives).

One PRS from the 9693_WGS_Sanger dataset (*t *= 2) was not found in the complete genome, nor were several PRS from the 9644_GSS_EMBL dataset. These probably represent non-*L. braziliensis *sequences, which are artefacts in the library. These reads were compared with GenBank nr/nt and are considered as heterologous sequences, probably derived from the cloning procedure and not removed before submission. In the 9693_WGS_Sanger dataset the spurious PRS matched with maxicircle (mitochondrial) sequences; the anomalous hits in the 9644_GSS_EMBL included 18 reads with no match in Genbank. Interestingly, the 18 sequences had a GC content of 20% as opposed to 58% for the genome of *L. braziliensis*.

As can also be seen in Table 2, 84 repetitions out of 500 PRSs were identified in the 9693_WGS_Sanger dataset with *t *= 1. These sequences occur between 3 and 5960 times in the assembled genome. This high amount is due to tandem repetitions. Three elements could be found in 30 to 32 different chromosomes (data not shown). On the other hand the amount of false positives is high: 376 (Table 2, (#no hit) and (# 1 hit)). In the 9644_GSS_EMBL dataset, 1289 putative elements were found (*t *= 1), but only 128 were found more than twice in the assembled genome, resulting in 1067 false positives. Interestingly, the value of the t parameter that minimizes the number of false positives is different for different datasets of the same genome: t > 6 for 9644_GSS_EMBL dataset and t = 4 for the two others.

### Checking the uniformity of read distribution

The high number of false positive PRSs in the 9644_GSS_EMBL dataset suggests that bias was introduced at some point, either in the library construction, sequencing procedure (for example, by re-sequencing clones) or sequence submission, causing data redundancy. The analysis of a multiple alignment of the reads containing the PRSs can be enlightening. If there are no mismatches, sequences are probably from the same genomic region, because genuine repeats show a lot of mismatches, mostly at the boundaries or flanking regions (See additional file 3: Align_PRS_1).

### Applying ReRep to data from new sequencing technologies

We further applied ReRep on a data set of *Escheria coli *obtained from a 454-read sequencing run. As this genome has been fully assembled, we were able to detect false positives and false negatives. Additional file 5 shows the repeats of minimum length of 50 bp in *E. coli *and their abundance. We applied ReRep with different sets of parameters, varying the *l *and the *t *parameter. For reasons of speed we increased the word size parameter to 18. Therefore, divergent repeat copies might not be found, but this is not a problem, as the assembler will be able to differentiate these repeats. Of the 23 repeats in the *E. coli *genome (Additional file 6), 14 occur more than twice, and the best parameter setting to minimize the number of false positives are *l *= 50 and *t *= 50, detecting 10 false positives, and 10 true positives. With *l *= 75 and *t *= 25, we detected 16 false positives and 15 repeats (9 of these are part of the 14 repeats that occur more often). The maximum amount of found repeats (true positives) was 19, (12 out of the 14 that occurs more than twice), at the cost of 105 false positives and 21 with the cost of 558 false positives.

## Discussion

The detection of repeats in GSS data is an open problem. Correct identification of repetitive elements greatly facilitates assembly, especially for organisms with a high proportion of repetitive sequences. In fact, most of the available eukaryotic genome sequences are not fully assembled, repeats being one of the main reasons. For instance, the *Trypanosoma cruzi *genome [15] could not be assembled because approximately 50% of its genomic content consists of repetitive sequences and low complexity regions, and also because of heterozygosis. Similar problems occur in the genome projects of human [16] and *Drosophila *[17]. Currently, most programs that detect repetitive elements are used with an assembled sequence. A few other approaches, such as [27], attempt to find repetitions in sequencing data, but work with high coverage data and do not propose an algorithm, but use the output of phrap and a general statistic of expected number of contigs.

Some assembler programs automatically mask k-mers that occur more frequently [28] or try to identify flanking regions or branching points [29] before and during the assembly process. Other approaches split contigs that have high coverage into several contigs after assembly, using SNPs [30].

In general, no analysis of repetitive sequences in early-stage GSS acquisition is performed, which often causes problems with assembly and evaluation. We have here presented a pipeline called ReRep (for "REads and REPeats") that can detect *de novo *repeats in GSS sequence data. In addition, a rough estimate of the total number of repeats in the genome can be obtained. The pipeline was evaluated by analyzing cosmid L9259 of *L. major *(Figure 2) and by applying ReRep to different datasets of *L. braziliensis *and to *E. coli*.

**Figure 3:**
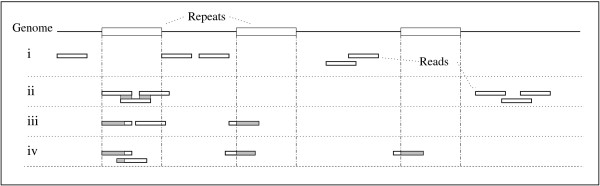
**Example of possible distributions of repetitive elements**. The top line represents a genome with three copies of the same repeat (white rectangular boxes). Smaller boxes represent reads. Gray parts of the reads indicate a repetition of this part of the read in the GSS dataset. Four different scenarios are shown: (i) No read (GSS) covers a repeat – no repetitive sequences can be found; (ii) one repeat is covered by three reads, but only the marked regions will occur twice; (iii) the beginning of two repeats is represented in the GSS dataset, but only this part can be found twice, from two different copies of the repeat; (iv) the initial parts of all repeats are covered, resulting in a partial coverage of the repeat.

Working with a dataset of low genome coverage poses certain difficulties. For a sequence to be identified as a repeat, it must occur at least twice in the dataset. Figure 3 shows possible scenarios for repeat identification. Not all repeats will be found, depending on the coverage and the distribution of the GSS data. Still, it is more likely to find short tandem repeats than, for example, LINEs or SINEs, because the short pattern will occur far more often in the dataset [6] and even more than once in a single read, as shown by the element TanPRS_1. The relative paucity of PRSs found in the 970_GSS dataset of *L. braziliensis *suggested a genome content of about 3% of repetitive elements. In the publication of the genome [7], around 10% of repeats was estimated.

Extrapolations to the whole genome based on the analysis of a dataset covering only 1.4% should be made with caution. The estimation after analysis of the 9693_WGS_Sanger dataset was more precise (6.6%), since the coverage was ten times higher.

Our pipeline includes the possibility of adjusting three parameters: i) the choice of the e-value and word size as a cut-off for sequence similarity detection allowing to find more divergent sequences; ii) the *l *parameter, which correlates with the expected size of the repeats and iii) the choice of the threshold *t*, which is related to the expected frequency of a base in an HSP, or the genome coverage in the sequencing project.

Table 2 shows the behaviour of different *t *parameters in three different datasets. Repetitive elements that are present with high frequency can be easily discriminated, but elements present in smaller numbers cannot be distinguished from genomic regions represented more than once in the GSS dataset. This phenomenon occurs even with low coverage, as evidenced by the fact that even with 16% coverage, 1335 contigs are formed in an assembly (CAP3 with 9693_WGS_Sanger dataset). Bias in library construction or in sequencing strategies and database submission makes correct assessment with ReRep more difficult. A correct assignment of PRS elements demands comparison of the ends of the repeats (flanking regions) and a careful examination of mismatches in a multiple alignment.

We normally choose the *l *parameter around half of the read length. This means that the overlap is at least 50%. Smaller *l *will find shorter repeats (Figure 2). In general, our algorithm does not have any limitations in terms of read length. But for short reads of around 35 bp, k-mers algorithms might be more efficient. The results from the analysis with data from 454 sequencing technology demonstrate this. These reads were also used to examine the false positive and false negative rates. For assembly purposes, it is better to set aside all detectable repetitive elements, even including a larger number of false positives.

But for repeat studies, the amount of false positives should be minimized. In principle, each overlap in an assembly that forms a contig can also be considered as a repetition. To avoid "detection" of these overlaps, the *t *parameter can be increased. Lander and Waterman [31] described the amount of overlaps of repeats by a poison distribution. So we know that, for a coverage of 1×, 10% of the genome is represented at least three times in the reads at hand. If the genome has repeats, this number will be higher, and less contigs will be formed. To distinguish true overlaps from repetitions, one can examine the border of the overlaps in the Sequence Landscape. If the amount of hits arises at a given position with at least two other reads, this represents probably the start of a repeat, as it is not likely that more than two sequence reads start on the position.

ReRep is an analytic tool for the analysis of GSS data before assembly, with the aim of estimating and identifying repetitive elements and their frequencies as precisely as possible, allowing good decisions to be made during assembly. Visualization tools like Consed [32] offer functionalities such as visualisation of the read depth, which has certain similarities with our SL. However, while the assembler tries to distinguish between these very similar reads as much as possible, ReRep tries to join and identify them as part of the same PRS. Assembly results cannot be parsed like the sequencing landscapes can after using different *l *or *t *parameters, because this information is not available. ReRep can find repetitions even in a small preliminary dataset, and the use of SLs provides a more quantitative and assessable way to analyze the presence of repetitive structures compared to homology matrices (Figure 2). Future work will include a better differentiation between real polymorphisms and sequencing errors using mate pair information, for instance.

## Conclusion

We have developed a new approach for determining repeats in GSS data. Depending on the genome coverage of the GSS dataset, it may not be possible to detect all repeats. But depending on the copy number of the repetitive elements identified, it is possible to obtain a rough estimate of their total frequency in the genome. In addition, the results can be used to optimize the sequence assembly process. After application of our pipeline to a *L. braziliensis *GSS dataset, four repeats were identified, of which three had not been documented so far.

## Methods

### Parasite strain and culture

*Leishmania braziliensis *(MHOM/BR/1968/M2904) cell mass was kindly provided by the Leishmania Reference Center at IOC, Fiocruz (Dr. E. Cupolillo). Promastigotes were cultured at 26°C in M199 medium (HyClone) supplemented with 10% (v/v) heat-inactivated fetal bovine serum (Gibco), 100 μM adenine, 10 μg/mL haemin, 40 μmM HEPES (N-2-hydroxyethylpiperazine-N-ethanesulfonic acid pH 7.4), penicillin 50 U/mL, streptomycin 50 μm/L and 2% human urine.

### Library construction and DNA sequencing

*L. braziliensis *high-molecular-weight genomic DNA was extracted by alkaline lysis, sheared and sized to around 2–3 kb to construct a genomic library in pUC18. Plasmid templates from the 2–3 kb insert semi-random library were prepared with a Wizard-SV 96 plasmid purification system (Promega). Double-stranded plasmid DNA templates were sequenced using Big Dye terminator chemistry with M13 forward and reverse primers and run on an ABI 3730 sequencer (Applied Biosystems).

### Datasets

We first constructed a dataset ('970_GSS') by sequencing 970 GSS with an average length of 550 bp and at least 150 bp of Phred quality equal to or higher than 20 [23]. All sequences were submitted to GenBank (Genbank: EI184570 – Genbank: EI185539). Assuming a 32 Mb haploid genome size for *L. braziliensis *[7], this represents a genome coverage of approximately 1.4%. A second dataset was constructed that included all available GSS for *L. braziliensis *data from EMBL ('9644_GSS_EMBL' EMBL: BX53013 – EMBL: BX53013; EMBL: BX897701 – EMBL:BX908718), comprising 9644 sequences (including the first dataset) with a average length of 544 bp. A third dataset consisted of the first 9693 reads (average length 539 bp) from the WGS dataset from the Sanger Institute ('9693_WGS_Sanger' [33]). We also used the 35 available chromosomes of *L. braziliensis *(EMBL: AM494938 to EMBL: AM494972) to better estimate the observed frequencies of the repetitive structures identified in this work. Due to gaps in the assembly, the exact number cannot be determined. A fourth dataset was constructed by simulating GSS data from the 35 chromosomes using a ReadSimulator (Huson, unpublished). This dataset ('9644_simGSS_Sanger') has approximately the same coverage (~16%), the same mean read length as the first dataset and approximately the same amount of base pairs as the other datasets. Pyrosequencing [34] (454) reads of *Escheria coli *K12 were obtained from [35]. We used around 400,000 reads (mean length of 109 bp), which cover the genome (4.6 MB) around 8 times.

### Known repeats and sequence features

A search for sequences similar to known repeats (Repbase volume 11 issue 12) was performed using the program Repeatmasker. In addition, we used Repeatmasker to find microsatellites, tandem repeats and low-complexity regions.

### *De novo *repeat identification

We implemented a pipeline (ReRep) that is based on similarity searches, the interpretation of sequence landscapes (SL), the assembly of clustered sequences and in-house Perl scripts. First, all reads are compared to each other with BLAST [19], with a word size of 8 and an e-value cut-off of 10^-20^, or NUCMER [20], with a word size of 11. Each result is pre-processed by joining overlapping hits and by deleting self-hits. For each read, an SL is constructed by counting how often each base of the read is part of a hit with another read. To generate the graph, we used the GD library [36]. The minimal length that an alignment must have to enter into the analysis was defined as *l*. Runs with different values for *l *can be performed.

### Sequence landscapes and repeat extension

The SL of each particular sequence represents how often its sub-sequences (with minimum length *l*) are represented in the dataset. All sub-sequences that occur more often than *t *times are retrieved from the SL as Putative Repetitive Sequences (PRS). The parameter *t *is determined according to the coverage.

To extend repeats that are longer than a GSS, the borders (the first or the last base) of the SL are analysed: If one or both borders score above or equal to the threshold *t*, the repeat is extended over the read. In this case, all reads that are part of the SL are assembled with CAP3 [21]. From the consensus of the assembly an SL is again constructed by repeating the comparison step. These steps are reiterated as long as the borders in the SL satisfy the threshold *t *or new GSS enter into the assembly. The identified repetitive structures, referred to in this work as Putative Repetitive Sequences (PRS), are obtained by retrieving each base pair with a frequency equal to or above the threshold *t *from the SL. Note that more than one PRS can be obtained from a single SL if two different regions of the SL score significantly. The consensus can be obtained by i) assembling all the reads forming the SL with CAP3 or ii) if parts of some reads are unique, a multiple alignment can be constructed using ClustalW [37]. To avoid reprocessing the SLs with known PRS, all sequences in the GSS data containing this PRS are masked by Cross_match (Green, unpublished). Besides masking known PRS, SLs containing this PRS sequence will be analyzed with a new value for *l *in the next iteration. Each PRS is then examined for tandem structures, repetitive substructures or fusion of more than one repeat.

### Repeat frequency determination

To count the frequency of each PRS we used the program 'Fuzznuc' of the EMBOSS package [25], allowing 0%, 5%, 10% and 15% of possible mismatches for short sequences. To compare this result with a similarity search result and to count the number of reads in which each PRS occurs, two BLAST comparisons (e-value 10^-20 ^and 10^-60^) were performed. A rough estimate of the abundance of the repetitive elements in the whole genome may be obtained by multiplying the frequency of each PRS in the GSS dataset by the reciprocal of its coverage.

## Availability and requirements

Project name: ReRep - Detecting repeats in sequencing reads

Project home page: 

Operating systems: Linux

Programming language: Perl

Other requirements: Perl GD, BLAST, Nucmer (MUMmer) and CAP3

License: ReRep is distributed under a GPL license

## Authors' contributions

TO, AB and WD designed the algorithm and wrote the manuscript. TO implemented and applied the algorithm. LG and MF obtained the 970 GSS sequences. All authors read and approved the final manuscript.

## Supplementary Material

Additional file 1 **Sequences and Sequence Landscapes of the PRS**. The DNA sequence and SL of PRS_1 – PRS_5 is given, using *l *= 400. Click here for file

Additional file 2 **PCR reaction for the elements PRS_1**. Column MW: 1 kb marker plus DNA Ladder. Size of the bands is indicated on the left. Column 1: amplification with primers: TTGTAAAACGACGGCCAGTG and CACACAGGAAACAGCTATGAC. Click here for file

Additional file 3 **Multiple alignment for PRS_1**. Multiple alignment of read GenBank: EI185111 and read GenBank: EI185194, representing PRS_1. Click here for file

Additional file 4 **Multiple alignment of the Tan-PRS_1 elements**. The tandem elements of PRS_1 were extracted from the original GSS. Elements A1-A11 were obtained from GenBank: EI185111; elements B1-B10 from GenBank: EI185194. Click here for file

Additional file 5 **Table of repeats of *E. coli *K12**. The name, the number of copies and the size of the repeats that are present in the *E. coli *K12 genome are listed, with indication, which of those are detected by ReRep analysis, with the corresponding parameter values of *l *and *t*. Click here for file

Additional file 6 FASTA file of repeats of *E. coli *K12. Click here for file
