# Supplementary material for: ReRep: Computational detection of repetitive sequences in genome survey sequences (GSS)
Source: BMC Bioinformatics. 2008 Sep 9;9:366. doi: 10.1186/1471-2105-9-366 (PMC2559850; doi:10.1186/1471-2105-9-366)
Supplement: Additional file 1 — Sequences and Sequence Landscapes of the PRS. The DNA sequence and SL of PRS_1 – PRS_5 is given, using l = 400. [file 1471-2105-9-366-S1.pdf]

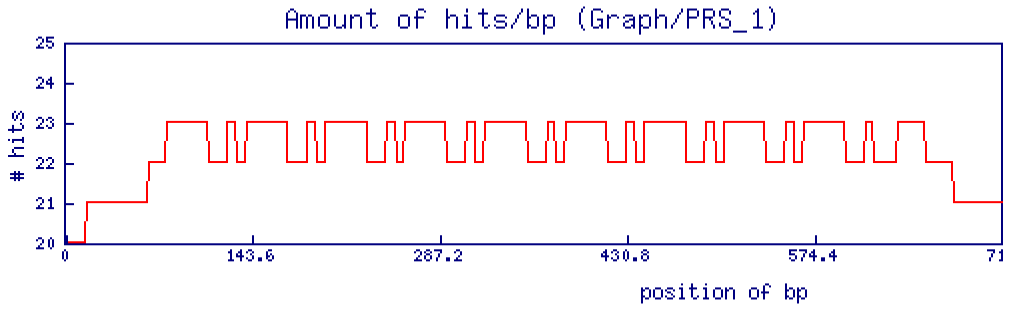
Amount of hits/bp (Graph/PRS_1)
# hits
25
24
23
22
21
20
0
143.6
287.2
430.8
574.4
71
position of bp

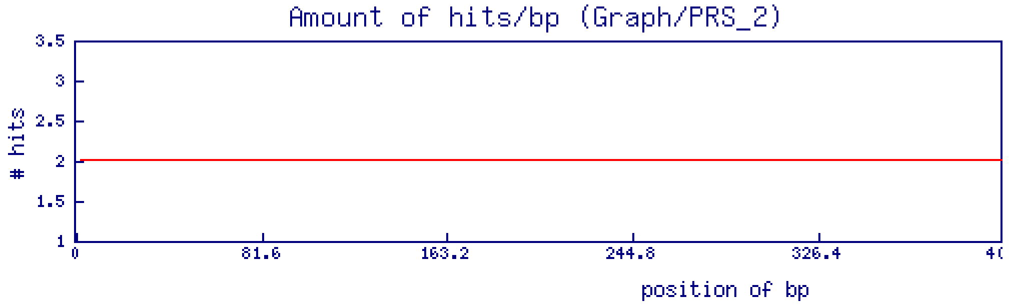
Amount of hits/bp (Graph/PRS_2)
# hits
3.5
3
2.5
2
1.5
1
0
81.6
163.2
244.8
326.4
40
position of bp

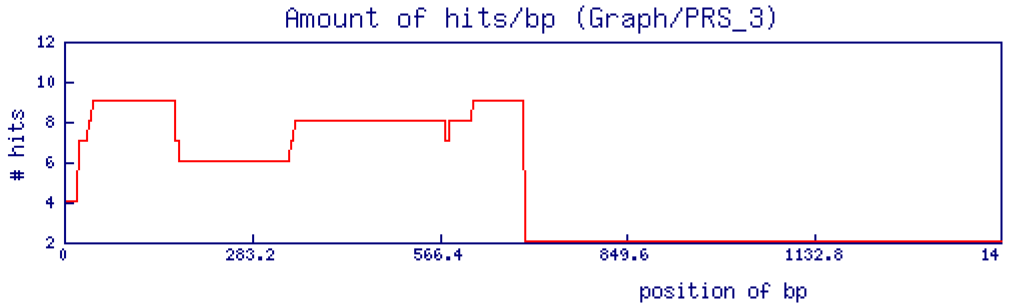
Amount of hits/bp (Graph/PRS_3)
# hits
12
10
8
6
4
2
0
283.2
566.4
849.6
1132.8
14
position of bp

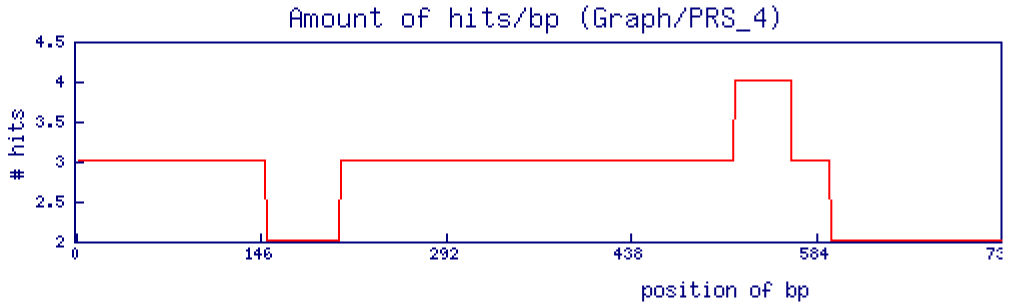
Amount of hits/bp (Graph/PRS_4)
# hits
4.5
4
3.5
3
2.5
2
0
146
292
438
584
73
position of bp

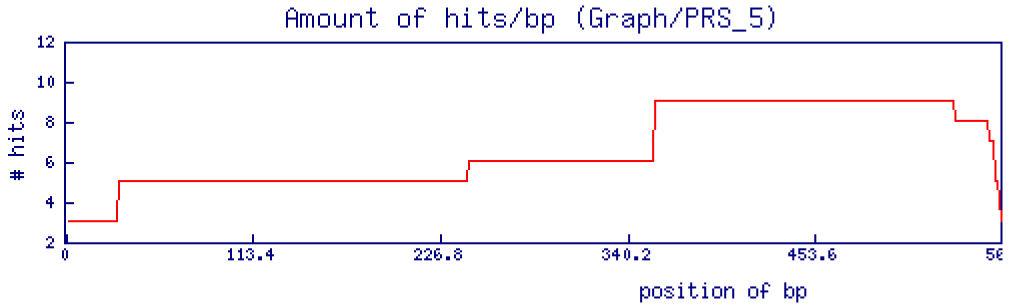
Amount of hits/bp (Graph/PRS_5)
# hits
12
10
8
6
4
2
0
113.4
226.8
340.2
453.6
56
position of bp

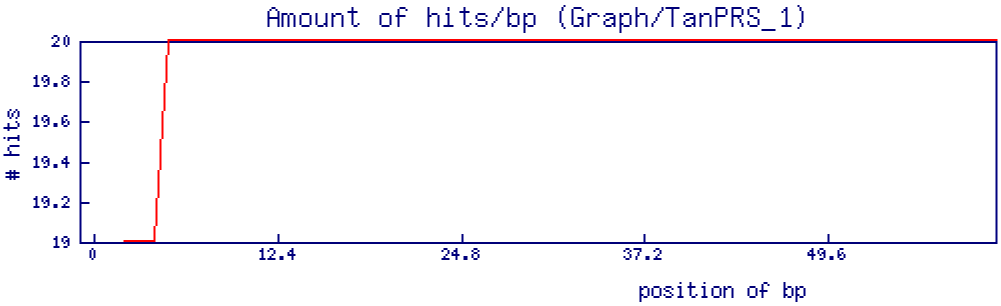


>PRS_1
GCTGCTTCTGACCCGTCTTGGTAGAGATGCGCAGCTGTGCAGTGTGGTGCTTCTGCCTTC
CGCTGCTTTTGACCCGTCTTGGTGGCGATGCGCAGCAGTGCAGTGTGGTGCTTCTGCGTT
CCGCTGCTTTTGACCCGTCTTGGTGGCGATGCGCAGCTGTGCAGTGTGGTGCTTCTGCGT
TCCGCTGCTTTTGACCCGTCTTGGTAGAGATGCGCAGCTGTGCAGTGTGGTGCTTCTGCG
TTCCGCTGCTTTTGACCCGTCTTGGTGGCGATGCGCAGCAGTGCAGTGTGGTGCTTCTGC
GTTCCGCTGCTTTTGACCCGTCTTGGTGGCGATGCGCAGCTGTGCAGTGTGGTGCTTCTG
CGTTCCGCTGCTTTTGACCCGTCTTGGTGGCGATGCGCAGCTGTGCAGTGTGGTGCTTCT
GCGTTCCGCTGCTTTTGACCCGTCTTGGTAGAGATGCGCAGCTGTGCAGTGTGGTGCTTC
TGCGTTCCGCTGCTTTTGACCCGTCTTGGTGGCGATGCGCAGCTGTGCAGTGTGGTGCTT
CTGCGTTCCGCTGCTTTTGACCCGTCTTGGTGGCGATGCGCAGCTGTGCAGTGTGGTGCT
TCTGCGTTCCGCTGCTTTTGACCCGTCTTGATAGAGATGCGCAGCTGTGCAGTGTGGTGC
TTCTGCGTTCCGCTGCTTTTGACCCGTCTTGGTGGCGATGCGCAGCTGTGCAGTGTG
>PRS_2
CACTTCTTGAAGTGGCACAGCGAAAAAAAACGACACC
GGCCATTCCCCCGGCGAAAGCAAAGCACACAGGAGAATGAAACGGGCAAGCAGGCATAGG
CGCCTCTTCTCGCTTATCCTTCGCGCTTGGGGCGAGTACACCACCCCGAAAACTCGAACA
CACCGGCGAAGCTGCCTTGCATCAACCCCCACAGCTATGCGAGCGGTGCGTGCGTGCGGG
GTTGGGCATCGGGCATGCGTTTGCGTTCGAGATTTGTTTCTCTTTCACCACACACCTTCA
CTTTGGTGCATTCTTCTGCTCCTCGCCCTGACGCACACTCTCCCCACACCCCACCCCCCA
TACACACACTTTCCTCGTTGGTGTCGCCGCCAGAGCTGAGCACACCGTGGCCAAGCACTA
CACAGCAAGA
>PRS_3
CTCTAGAGGATCTCCAAGAGGTAATGATGAACAGAGAGAGAGAGAGAGAGGCGAAGACAT
TCACGCGCATAGAGACACGGCAACAACATCAAAGAACAAGCCAAAAAACAAAATGACACG
ACAAACGTGTGTCGAAACAGCAAAGCAATGGAGGGGAGGGGGAGGGGGACGTGAAAGGGG
TAAAGTTGCTTCTCTGCCATATGTGTGCGCGTGTGTCTTCTCCTGTTTTTTTTTTTTGCG
CAAGGACGAGAAAGGATGGCAGAGACCCATAAAGGCTCTGCACCCCCCCCCCACACACA
CACACGTACGGGCAGTTCAAAGGGTTTGTAGAGTGGCCTGTGTGTGTGTGTGTGGGGGGG
GTGCTCGTAGGGGAGAATTCCCGTTGTCGGAGGCCTTACGTTGTTGATTAGCTTGTTTTC
CTGTCGCTGGGAGGTTTATGAAGGCGCGCAGCAGACAAGGAAGTCGGCAAGGCAAAGCAA
AAGCACACAGAGAGCGATCATGGTGAATGATGTATAAGGAAGGGCGTGTCAGGAAGGAAG
GAAGGAGGGAAAGAGGGAAGGAGGAAAGGAAGGAAGGAAGGGGGTGTGGGCGAAGAGTAA
GAAGCGAAAAAGTAACTTTCAAGCTAAGGAGTCAAAAGTGCGCATGACAAGAGAAGAGCA
AAGAAAAGGTAAGAGAGAGAGGGGGGGGGGGGAGGTTAGTGGAGGCGAGAGCAAAAGAAA
GGGTGTTATTGTGTGAAAGGAGGCTTGGGTAGATGGAGAGAGCAAGCAGAGCCAAAAAGA
AACCAATGCGGGGTAGCGAGGGGACAAGTACGAGCACCCCATCCTGGCATTCCCGCCCGC
CTTCCTCTCCCTCGTCCTCAGCACGCGGGTACTCGGGATAGATACAGGAAAGGAAGAGCG
AGGGGTGAAGGGAGGAAAGAGAGGTAGCTTAGTAAGGAGAGATGGCTGGGGGAAGTGGGA
CGAAGAGGCTACAGCAAGTATACGGAGAGATGTTACACAATAAAGAAAATCAAATCCCCC
ACGGGCCACGTGAGGCTCACAACGAGACAAAAGACAACCAGAAGAAGGCATGCGGGTAGG
GGGGAGGAGAGAAGGAAGTATCAGCAAGGAGAGAGACAGAAGAAAGGAAACACACAAAGA
GATGAGAGATGGGAAGGAGTAGGACGCAAACGCCGAAGCCGGAAAGAAACCAAACAAAAC
ACGAAAGAAGTGAGGCCGATAATACGACAAGCGAGGAGGAGAGAAAAAAGAGACGCCGAG
GGAAGAGAGAAGAAACAGGGGGTAAGGGGAGAGGATGACAGCAAGGAATTCGAGATGGAG
GGCGCCAAGACCGATGAGGGTGCAACCGCAAAGCACCTCGAGTTCACTGGCGCGAGTGGT
GTGATGCTCTTAGCATGCGGCGCTGGTGCTTAGTA
>PRS_4
GTGACGGAAGCTGAGCGGCTCCCGCTGCGGCTGACGAGGGCCTCCGCTAGCGTGAAACGG

AACGAGCAGGACGACTGGGTGCGCGGTGCCGCTGTAGTGTGCCCCTGTGCCGACGGCCAT
CGCAGCCCACGCGCGTCGGAATGGCCTTTGGCTGCTTTGTTTCTCCCTCGCTCGTCGCCT
CTCGGTGACGGCGTCGCCATTGCGAATTCAGAGTCTGATGGATGCGTGTGTTGGGGGCTC
TCTCCCTCTCTCGCAGCCCCCACCACCCCCTCCCTTTCCGTTGCTTCTCTTTTCCTATTG
CTGCGCGGGTGTGCGTGCCGCTGGTAGACACCTTGAGCTCCTGGCCATTCGCCCTGCAGT
GGCGCAGACGACCATCAAGGTCATGGGGTGGGCCTCGACACACCCCGCTGTCCCACGGCG
AGCACACCGCGCGTGTCTGCTTTCCTTTGCTTTCTGTATTCTGTGCTTTCGACTCTGCAA
ACGCCGTGCGGCGGGCGCAGGCGGCCTGATGTGCGCCCTCTCTCTCTCTCGGCAGCGCGA
CACGGGGAATGGACGTGAGCCAGAGGGGAGGAAGGCGAGCACGCGCCCCTGCGCCATGGC
CGGCAGTCGTGCGCTTCCTTCTCGCGACCTCCCAGAGAGTCACCCTCTCCCCCGCCCAGC
ACGCGTCAGCAGGGCGCCCTCGCCTACGGCACTCGCCGTCCTCCGACACGCGCCCTATTC
GGGGCTGGA
>PRS_5
GCATGGGTTCCCGATTGTTCCCAGCGATCCCTTCGGGTTCTGTACCTTCCGTTTTTGTAG
ATTTGCCGACCCCAACGCCCCCGGGATTCGTGTACGTGCGTCCCTGCGTGCCCCCCTCCC
CGCGGGCGCTTGTAGCTTGATTGTTTTTTCGGCGGCTGACCGACTCGTTTCCTTTCTTCT
GGCGACTCCCCGCATGTCCGTTTGGCATGCCGCCCTCTGACCGTAATGAATGGGCACCCG
GTGGGGGAGGCTTCCCCGGTACCGGTGCCAAGCCATAAGAAAAAGCACACAAAAGAAGTC
CAAGACGACTTGATGTGCTCCTCACGCGACCGAGCGCGGTAGAGGGGACCGGCGGCATAA
TCATGACGCGGCGCCGCGGTCGTGGTTGCGTGCCTGCCTTGCCGCTTCCCAATGGGGTCG
CGAAGACTCTCTCCATACCCATCTGCCTTCCACAAATCTTTCTCCCTTCTCGCCCTCTAC
TTGTCTTCAAGAAATCATATCTTCGCGAGCACCCTATCACACAGGACGGTGGCATTAGGG
TTAGGGTTAGGGTTAGGGTTAGGGTT
>TanPRS_1
TTCCGCTGCTTTTGACCCGTCTTGGTGGCGATGCGCAGCTGTGCAGTGTGGTGCTTCTGCG
