# Supplementary material for: ReRep: Computational detection of repetitive sequences in genome survey sequences (GSS)
Source: BMC Bioinformatics. 2008 Sep 9;9:366. doi: 10.1186/1471-2105-9-366 (PMC2559850; doi:10.1186/1471-2105-9-366)
Supplement: Additional file 2 — PCR reaction for the elements PRS_1. Column MW: 1 kb marker plus DNA Ladder. Size of the bands is indicated on the left. Column 1: amplification with primers: TTGTAAAACGACGGCCAGTG and CACACAGGAAACAGCTATGAC. [file 1471-2105-9-366-S2.pdf]

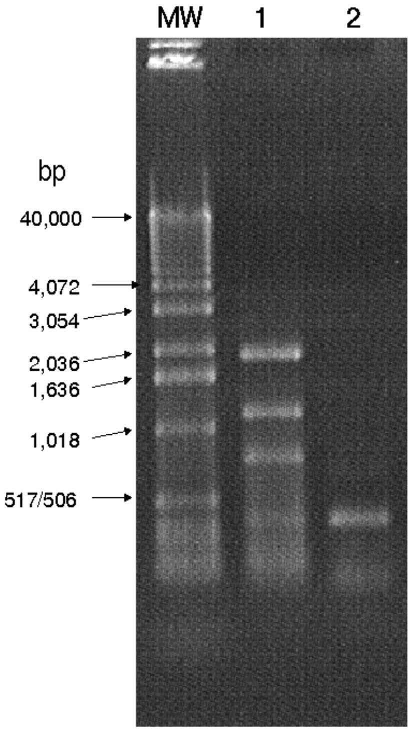

Supplemental Figure 4: PCR reaction for the elements PRS_1 in *Leishmania braziliensis*. Column MW: 1kb marker Plus DNA Ladder Raia. Size of the bands is indicated on the left. Column 1: amplification with primers: TTGTAAAACGACGGCCAGTG and CACACAGGAAACAGCTATGAC.
