# Supplementary material for: ReRep: Computational detection of repetitive sequences in genome survey sequences (GSS)
Source: BMC Bioinformatics. 2008 Sep 9;9:366. doi: 10.1186/1471-2105-9-366 (PMC2559850; doi:10.1186/1471-2105-9-366)
Supplement: Additional file 3 — Multiple alignment for PRS_1. Multiple alignment of read GenBank: EI185111 and read GenBank: EI185194, representing PRS_1. [file 1471-2105-9-366-S3.pdf]

Alignment: PRS_1.aln
Seaview [blocks=10 fontsize=10 A4] on Wed Mar 7 08:00:53 2007

```
          1
LWB1453   CACACTGCAC AGCTGCGCAT CGCCACCAAG ACGGGTCAAA AGCAGCGGAA CGCAGAAGCA CCACACTGCA
LBW1526   ---------- ---------- ---------- ---------A AGCAGCGGAA CGCAGAAGCA CCACACTGCA

         71
LWB1453   CAGCTGCGCA TCTCTACCAA GACGGGTCAA AAGCAGCGGA ACGCAGAAGC ACCACACTGC ACAGCTGCGC
LBW1526   CAACTGCGCA TCGCCATCAA GACGGGTCAA AAGCAGCGGA ACGCAGAAGC ACCACACTGC ACAGCTGCGC

        141
LWB1453   ATCGCCACCA AGACGGGTCA AAAGCAGCGG AACGCAGAAC CACCACACTG CACAGCTGCG CATCGCCACC
LBW1526   ATCGCCACCA AGACGGGTCA AAAGCAGCGG AACGCAGAAG CACCACACTG CACAGCTGCG CATCGCCACC

        211
LWB1453   AAGACGGGTC AAAAGCAGCG GAACGCAGAA GCACCACACT GCACAACTGC GCATCGCCAC CAAGACGGGT
LBW1526   AAGACGGGTC AAAAGCAGCG GAACGCAGAA GCACCACACT GCACAGCTGC GCATCTCTAC CAAGACGGGT

        281
LWB1453   CAAAAGCAGC GGAACGCAGA AGCACCACAC TGCACAGCTG CGCATCGCCA CCAAGACGGG TCAAAAGCAG
LBW1526   CAAAAGCAGC GGAACGCAGA AGCACCACAC TGCACAGCTG CGCATCGCCA CCAAGACGGG TCAAAAGCAG

        351
LWB1453   CGGAACGCAG AAGCACCACA CTGCACAGCT GCGCATCGCC ACCAAGACGG GTCAAAAGCA GCGGAACGCA
LBW1526   CGGAACGCAG AACCACCACA CTGCACAGCT GCGCATCGCC ACCAAGACGG GTCAAAAGCA GCGGAACGCA

        421
LWB1453   GAAGCACCAC ACTGCACTGC TGCGCATCGC CACCAAGACG GGTCAAAAGC AGCGGAACGC AGAAGCACCA
LBW1526   GAAGCACCAC ACTGCACAAC TGCGCATCGC CACCAAGACG GGTCAAAAGC AGCGGAACGC AGAAGCACCA

        491
LWB1453   CACTGCACAG CTGCGCATCT CTACCAAGAC GGGTCAAAAG CAGCGGAACG CAGAAGCACC ACACTGCACA
LBW1526   CACTGCACAG CTGCGCATCG CCACCAAGAC GGGTCAAAAG CAGCGGAACG CAGAAGCACC ACACTGCACA

        561
LWB1453   GCTGCGCATC GCCACCAAGA CGGGTCAAAA GCAGCGGAAC GCAGAACCAC CACACTGCAC TGCTGCGCAT
LBW1526   GCTGCGCATC GCCACCAAGA CGGGTCAAAA GCAGCGGAAC GCAGAAGCAC CACACTGCAC TGCTGCGCAT

        631
LWB1453   CGCCACCAAG ACGGATCAAA AGCAGCGAAA GGCAGAAGCA CCACACTGCA CAACTGCGCA TCGCCACCAA
LBW1526   CGCCACCAAG ACGGGTCAAA AGCAGCGGAA CGCAGAAGCA CCACACTGCA CAGCTGCGCA TCTCTACCAA

        701
LWB1453   GAC------- -------
LBW1526   GACGGGTCAG AAGCAGC
```
