# Supplementary material for: ReRep: Computational detection of repetitive sequences in genome survey sequences (GSS)
Source: BMC Bioinformatics. 2008 Sep 9;9:366. doi: 10.1186/1471-2105-9-366 (PMC2559850; doi:10.1186/1471-2105-9-366)
Supplement: Additional file 4 — Multiple alignment of the Tan-PRS_1 elements. The tandem elements of PRS_1 were extracted from the original GSS. Elements A1-A11 were obtained from GenBank: EI185111; elements B1-B10 from GenBank: EI185194. [file 1471-2105-9-366-S4.pdf]

Alignment: TanPRS_1.aln
Seaview [blocks=10 fontsize=10 A4] on Wed Mar 7 08:07:11 2007

```
       1
A4     TTCCGCTGCT TTTGACCCGT CTTGGTGGCG ATGCGCAGCA GTGCAGTGTG GTGCTTCTGC G
B1     TTCCGCTGCT TTTGACCCGT CTTGGTGGCG ATGCGCAGCA GTGCAGTGTG GTGCTTCTGC G
A11    TTCCGCTGCT TTTGACCCGT CTTGGTGGCG ATGCGCAGCT GTGCAGTGTG ---------- -
A8     TTCCGCTGCT TTTGACCCGT CTTGGTGGCG ATGCGCAGCT GTGCAGTGTG GTGGTTCTGC G
B5     TTCCGCTGCT TTTGACCCGT CTTGGTGGCG ATGCGCAGCT GTGCAGTGTG GTGGTTCTGC G
A10    TTCCGCTGCT TTTGACCCGT CTTGGTAGAG ATGCGCAGCT GTGCAGTGTG GTGCTTCTGC G
A3     TTCCGCTGCT TTTGACCCGT CTTGGTAGAG ATGCGCAGCT GTGCAGTGTG GTGCTTCTGC G
B7     TTCCGCTGCT TTTGACCCGT CTTGGTAGAG ATGCGCAGCT GTGCAGTGTG GTGCTTCTGC G
A7     TTCCGCTGCT TTTGACCCGT CTTGGTGGCG ATGCGCAGTT GTGCAGTGTG GTGCTTCTGC G
B4     TTCCGCTGCT TTTGACCCGT CTTGGTGGCG ATGCGCAGTT GTGCAGTGTG GTGCTTCTGC G
B10    TTCCGCTGCT TTTGACCCGT CTTGATGGCG ATGCGCAGTT GTGCAGTGTG GTGCTTCTGC G
A2     TTCCGCTGCT TTTGACCCGT CTTGGTGGCG ATGCGCAGCT GTGCAGTGTG GTGCTTCTGC G
A5     TTCCGCTGCT TTTGACCCGT CTTGGTGGCG ATGCGCAGCT GTGCAGTGTG GTGCTTCTGC G
A6     TTCCGCTGCT TTTGACCCGT CTTGGTGGCG ATGCGCAGCT GTGCAGTGTG GTGCTTCTGC G
A9     TTCCGCTGCT TTTGACCCGT CTTGGTGGCG ATGCGCAGCT GTGCAGTGTG GTGCTTCTGC G
B2     TTCCGCTGCT TTTGACCCGT CTTGGTGGCG ATGCGCAGCT GTGCAGTGTG GTGCTTCTGC G
B3     TTCCGCTGCT TTTGACCCGT CTTGGTGGCG ATGCGCAGCT GTGCAGTGTG GTGCTTCTGC G
B6     TTCCGCTGCT TTTGACCCGT CTTGGTGGCG ATGCGCAGCT GTGCAGTGTG GTGCTTCTGC G
B8     TTCCGCTGCT TTTGACCCGT CTTGGTGGCG ATGCGCAGCT GTGCAGTGTG GTGCTTCTGC G
B9     TTCCGCTGCT TTTGACCCGT CTTGGTGGCG ATGCGCAGCT GTGCAGTGTG GTGCTTCTGC G
A1     TTTCGCTGCT TTTGATCCGT CTTGGTGGCG ATGCGCAGCA GTGCAGTGTG GTGGTTCTGC G
```
