# Supplementary material for: ReRep: Computational detection of repetitive sequences in genome survey sequences (GSS)
Source: BMC Bioinformatics. 2008 Sep 9;9:366. doi: 10.1186/1471-2105-9-366 (PMC2559850; doi:10.1186/1471-2105-9-366)
Supplement: Additional file 5 — Table of repeats of E. coli K12. The name, the number of copies and the size of the repeats that are present in the E. coli K12 genome are listed, with indication, which of those are detected by ReRep analysis, with the corresponding parameter values of l and t. [file 1471-2105-9-366-S5.pdf]

454 Analysis

Name	No. copies	Length	l 75 t 25	l 75 t 15	l 50 t 50	l 50 t 25	l 25 t 25
R1	2	5419	X	X	X	X	X
R2	5	129	X	X		X	X
R3	3	1254	X	X		X	X
R4	7	967	X	X	X	X	X
R5	6	1335	X	X	X	X	X
R6	5	1272	X	X	X	X	X
R7	2	1080		X		X	X
R8	19	110	X	X	X	X	X
R9	2	262		X		X	X
R10	2	242					
R11	2	355		X			
R12	2	85		X			X
R13	3	2561	X	X		X	X
R14	4	76				X	X
R15	4	104		X		X	X
R16	4	77	X	X		X	X
R17	11	1206	X	X	X	X	X
R18	6	5441	X	X	X	X	X
R19	4	5351	X	X	X	X	X
R20	4	89					X
R21	3	5689	X	X	X	X	X
R22	2	1244	X	X		X	X
R23	3	5322	X	X	X	X	X
False Positive			16	151	10	105	558
