# Supplementary material for: ReRep: Computational detection of repetitive sequences in genome survey sequences (GSS)
Source: BMC Bioinformatics. 2008 Sep 9;9:366. doi: 10.1186/1471-2105-9-366 (PMC2559850; doi:10.1186/1471-2105-9-366)
Supplement: Additional file 6 — FASTA file of repeats of E. coli K12. [file 1471-2105-9-366-S6.pdf]

>R1
GCTTAATTTGATGCCTGGCAGTTCCCTACTCTCGCATGGGGAGACCCCACACTACCATCG
GCGCTACGGCGTTTCACTTCTGAGTTCGGCATGGGGTCAGGTGGGACCACCGCGCTACGG
CCGCCAGGCAAATTCTGTTTTATCAGACCGCTTCTGCGTTCTGATTTAATCTGTATCAGG
CTGAAAATCTTCTCTCAATCCGCCAAAACAGCTTCGGCGTTGTAAGGTTAAGCCTCACGG
TTCATTAGTACCGGTTAGCTCAACGCATCGCTGCGCTTACACACCCGGCCTATCAACGTC
GTCGTCTTCAACGTTCCTTCAGGACCCTCAAAGAGTCAGGGAGAACTCATCTCGGGGCAA
GTTTCGTGCTTAGATGCTTTCAGCACTTATCTCTTCCGCATTTAGCTACCGGGCAGTGCC
ATTGGCATGACAACCCGAACACCAGTGATGCGTCCACTCCGGTCCTCTCGTACTAGGAGC
AGCCCCCCTCAGTTCTCCAGCGCCCACGGCAGATAGGGACCGAACTGTCTCACGACGTTC
TAAACCCAGCTCGCGTACCACTTTAAATGGCGAACAGCAACACCCTTGGGACCTACTTCA
GCCCCAGGATGTGATGAGCCGACATCGAGGTGCCAAACACCGCCGTCGATATGAACTCTT
GGGCGGTATCAGCCTGTTATCCCCGGAGTACCTTTTATCCGTTGAGCGATGGCCCTTCCA
TTCAGAACCACCGGATCACTATGACCTGCTTTCGCACCTGCTCGCGCCGTCACGCTCGCA
GTCAAGCTGGCTTATGCCATTGCACTAACCTCCTGATGTCCGACCAGGATTAGCCAACCT
TCGTGCTCCTCCGTTACTCTTTAGGAGGAGACCGCCCCAGTCAAACTACCCACCAGACAC
TGTCCGCAACCCGGATTACGGGTCAACGTTAGAACATCAAACATTAAAGGGTGGTATTTC
AAGGTCGGCTCCATGCAGACTGGCGTCCACACTTCAAAGCCTCCCACCTATCCTACACAT
CAAGGCTCAATGTTCAGTGTCAAGCTATAGTAAAGGTTCACGGGGTCTTTCCGTCTTGCC
GCGGGTACACTGCATCTTCACAGCGAGTTCAATTTCACTGAGTCTCGGGTGGAGACAGCC
TGGCCATCATTACGCCATTCGTGCAGGTCGGAACTTACCCGACAAGGAATTTCGCTACCT
TAGGACCGTTATAGTTACGGCCGCCGTTTACCGGGGCTTCGATCAAGAGCTTCGCTTGCG
CTAACCCCATCAATTAACCTTCCGGCACCGGGCAGGCGTCACACCGTATACGTCCACTTT
CGTGTTTGCACAGTGCTGTGTTTTTAATAAACAGTTGCAGCCAGCTGGTATCTTCGACTG
ATTTCAGCTCCACGAGCAAGTCGCTTCACCTACATATCAGCGTGCCTTCTCCCGAAGTTA
CGGCACCATTTTGCCTAGTTCCTTCACCCGAGTTCTCTCAAGCGCCTTGGTATTCTCTAC
CTGACCACCTGTGTCGGTTTGGGGTACGATTTGATGTTACCTGATGCTTAGAGGCTTTTC
CTGGAAGCAGGGCATTTGTTGCTTCAGCACCGTAGTGCCTCGTCATCACGCCTCAGCCTT
GATTTTCCGGATTTGCCTGGAAAACCAGCCTACACGCTTAAACCGGGACAACCGTCGCCC
GGCCAACATAGCCTTCTCCGTCCCCCCTTCGCAGTAACACCAAGTACAGGAATATTAACC
TGTTTCCCATCGACTACGCCTTTCGGCCTCGCCTTAGGGGTCGACTCACCCTGCCCCGAT
TAACGTTGGACAGGAACCCTTGGTCTTCCGGCGAGCGGGCTTTTCACCCGCTTTATCGTT
ACTTATGTCAGCATTCGCACTTCTGATACCTCCAGCATACCTCACAGCACACCTTCACAG
GCTTACAGAACGCTCCCCTACCCAACAACGCATAAGCGTCGCTGCCGCAGCTTCGGTGCA
TGGTTTAGCCCCGTTACATCTTCCGCGCAGGCCGACTCGACCAGTGAGCTATTACGCTTT
CTTTAAATGATGGCTGCTTCTAAGCCAACATCCTGGCTGTCTGGGCCTTCCCACATCGTT
TCCCACTTAACCATGACTTTGGGACCTTAGCTGGCGGTCTGGGTTGTTTCCCTCTTCACG
ACGGACGTTAGCACCCGCCGTGTGTCTCCCGTGATAACATTCTCCGGTATTCGCAGTTTG
CATCGGGTTGGTAAGTCGGGATGACCCCCTTGCCGAAACAGTGCTCTACCCCCGGAGATG
AATTCACGAGGCGCTACCTAAATAGCTTTCGGGGAGAACCAGCTATCTCCCGGTTTGATT
GGCCTTTCACCCCCAGCCACAAGTCATCCGCTAATTTTTCAACATTAGTCGGTTCGGTCC
TCCAGTTAGTGTTACCCAACCTTCAACCTGCCCATGGCTAGATCACCGGGTTTCGGGTCT
ATACCCTGCAACTTAACGCCCAGTTAAGACTCGGTTTCCCTTCGGCTCCCCTATTCGGTT
AACCTTGCTACAGAATATAAGTCGCTGACCCATTATACAAAAGGTACGCAGTCACACGCC
TAAGCATGCTCCCACTGCTTGTACGTACACGGTTTCAGGTTCTTTTTCACTCCCCTCGCC
GGGGTTCTTTTCGCCTTTCCCTCACGGTACTGGTTCACTATCGGTCAGTCAGGAGTATTT
AGCCTTGGAGGATGGTCCCCCCATATTCAGACAGGATACCACGTGTCCCGCCCTACTCAT
CGAGCTCACAGCATGTGCATTTTTGTGTACGGGGCTGTCACCCTGTATCGCGCGCCTTTC

CAGACGCTTCCACTAACACACACACTGATTCAGGCTCTGGGCTCCTCCCCGTTCGCTCGC
CGCTACTGGGGGAATCTCGGTTGATTTCTTTTCCTCGGGGTACTTAGATGTTTCAGTTCC
CCCGGTTCGCCTCATTAACCTATGGATTCAGTTAATGATAGTGTGTCGAAACACACTGGG
TTTCCCCATTCGGAAATCGCCGGTTATAACGGTTCATATCACCTTACCGACGCTTATCGC
AGATTAGCACGTCCTTCATCGCCTCTGACTGCCAGGGCATCCACCGTGTACGCTTAGTCG
CTTAACCTCACAACCCGAAGATGTTTCATGCGATCCATCATCGAGTTGCGAAAATTTGAG
AGACTCACGAACAACTTTCATTGTTCAGTGTTTCAATTTTCAGCTTGATCCAGATTTTTA
AAGAGCAAAACTTCGCAGTGAACCTTTGCAGGTACACTCTGAAGTATTTTTTATTTAACC
ACTACAGAGATGGTGGAGCTATGCGGGATCGAACCGCAGACCTCCTGCGTGCAAAGCAGG
CGCTCTCCCAGCTGAGCTATAGCCCCATAACATGTAGTTAAAACCTCTTCAAATTTGCCG
TGCAAATTTGGTAGGCCTGAGTGGACTTGAACCACCGACCTCACCCTTATCAGGGGTGCG
CTCTAACCACCTGAGCTACAAGCCTGTAGAGGTTTTACTGCTCATTTTCATCAGACAATC
TGTGTGAGCACTGCAAAGTACGCTTCTTTAAGGTAAGGAGGTGATCCAACCGCAGGTTCC
CCTACGGTTACCTTGTTACGACTTCACCCCAGTCATGAATCACAAAGTGGTAAGCGCCCT
CCCGAAGGTTAAGCTACCTACTTCTTTTGCAACCCACTCCCATGGTGTGACGGGCGGTGT
GTACAAGGCCCGGGAACGTATTCACCGTGGCATTCTGATCCACGATTACTAGCGATTCCG
ACTTCATGGAGTCGAGTTGCAGACTCCAATCCGGACTACGACGCACTTTATGAGGTCCGC
TTGCTCTCGCGAGGTCGCTTCTCTTTGTATGCGCCATTGTAGCACGTGTGTAGCCCTGGT
CGTAAGGGCCATGATGACTTGACGTCATCCCCACCTTCCTCCAGTTTATCACTGGCAGTC
TCCTTTGAGTTCCCGGCCGGACCGCTGGCAACAAAAGATAAGGGTTGCGCTCGTTGCGGG
ACTTAACCCAACATTTCACAACACGAGCTGACGACAGCCATGCAGCACCTGTCTCACAGT
TCCCGAAGGCACAAACCCATCTCTGAAAACTTCCGTGGATGTCAAGACCAGGTAAGGTTC
TTCGCGTTGCATCGAATTAAACCACATGCTCCACCGCTTGTGCGGGCCCCCGTCAATTCA
TTTGAGTTTTAACCTTGCGGCCGTACTCCCCAGGCGGTCGACTTAACGCGTTAGCTCCGG
AAGCCACGCCTCAAGGGCACAACCTCCAAGTCGACATCGTTTACGGCGTGGACTACCAGG
GTATCTAATCCTGTTTGCTCCCCACGCTTTCGCACCTGAGCGTCAGTCTTCGTCCAGGGG
GCCGCCTTCGCCACCGGTATTCCTCCAGATCTCTACGCATTTCACCGCTACACCTGGAAT
TCTACCCCCCTCTACGAGACTCAAGCTTGCCAGTATCAGATGCAGTTCCCAGGTTGAGCC
CGGGGATTTCACATCTGACTTAACAAACCGCCTGCGTGCGCTTTACGCCCAGTAATTCCG
ATTAACGCTTGCACCCTCCGTATTACCGCGGCTGCTGGCACGGAGTTAGCCGGTGCTTCT
TCTGCGGGTAACGTCAATGAGCAAAGGTATTAACTTTACTCCCTTCCTCCCCGCTGAAAG
TACTTTACAACCCGAAGGCCTTCTTCATACACGCGGCATGGCTGCATCAGGCTTGCGCCC
ATTGTGCAATATTCCCCACTGCTGCCTCCCGTAGGAGTCTGGACCGTGTCTCAGTTCCAG
TGTGGCTGGTCATCCTCTCAGACCAGCTAGGGATCGTCGCCTAGGTGAGCCGTTACCCCA
CCAACAAGCTAATCCCATCTGGGCACATCCGATGGCAAGAGGCCCGAAGGTCCCCCTCTT
TGGTCTTGCGACGTTATGCGGTATTAGCTACCGTTTCCAGTAGTTATCCCCCTCCATCAG
GCAGTTTCCCAGACATTACTCACCCGTCCGCCACTCGTCAGCAAAAAAGCAAGCTGCTTC
CTGTTACCGTTCGACTTGCATGTGTTAGGCCTGCCGCCAGCGTTCAATCTGAGCCATGAT
CAAACTCTTCAATTTAAAAGTTTGATGCTCAAAGAATTAAACTTCGTAATGAATTACGTG
TTCACTCTTGAGACTTGGTATTCATTTTTCGTCTTGCGACGTTAAGAATCCGTATCTTCG
AGTGCCCACACAGATTGTCTGATAAATTGTTAAAGAGCAGTTGCGACGCGGCTTTCAGCT
CACTGTCGCGAGGTGGCGTATATTACGCTTTCCTCTTTCAGAGTCAACCCTGAATTTCAG
GATTTTTCTCTTCAACCGAACCGGCTGTTTGTGTGAAGTGATTCACATCCGCCGTGTCGA
TGGAGGCGCATTATAGGGA
>R2
TTATACTCGTCATACTTCAAGTTGCATGTGCTGCGTCTGCGTTCGCTCACCCCAGTCACT
TACTTATGTAAGCTCCTGGGGATTCACTCGCTTGTCGCCTTCCTGCAACTCGAATTATTT
AGAGTATAT

>R3
ATATTAAAAAAAGCAACTGTAGATTCAATTGGTCAACGCAACAGTTATGTGAAAACATGG
GGTTGCGGAGGTTTTTTGAATGAGACGAACATTTACAGCAGAGGAAAAAGCCTCTGTTTT
TGAACTATGGAAGAACGGAACAGGCTTCAGTGAAATAGCGAATATCCTGGGTTCAAAACC
CGGAACGATCTTCACTATGTTAAGGGATACTGGCGGCATAAAACCCCATGAGCGTAAGCG
GGCTGTAGCTCACCTGACACTGTCTGAGCGCGAGGAGATACGAGCTGGTTTGTCAGCCAA
AATGAGCATTCGTGCGATAGCTACTGCGCTGAATCGCAGTCCTTCGACGATCTCACGTGA
AGTTCAGCGTAATCGGGGCAGACGCTATTACAAAGCTGTTGATGCTAATAACCGAGCCAA
CAGAATGGCGAAAAGGCCAAAACCGTGCTTACTGGATCAAAATTTACCATTGCGAAAGCT
TGTTCTGGAAAAGCTGGAGATGAAATGGTCTCCAGAGCAAATATCAGGATGGTTAAGGCG
AACAAAACCACGTCAAAAAACGCTGCGAATATCACCTGAGACAATTTATAAAACGCTGTA
CTTTCGTAGCCGTGAAGCGCTACACCACCTGAATATACAGCATCTGCGACGGTCGCATAG
CCTTCGCCATGGCAGGCGTCATACCCGCAAAGGCGAAAGAGGTACGATTAACATAGTGAA
CGGAACACCAATTCACGAACGTTCCCGAAATATCGATAACAGACGCTCTCTAGGGCATTG
GGAGGGCGATTTAGTCTCAGGTACAAAAAACTCTCATATAGCCACACTTGTAGACCGAAA
ATCACGTTATACGATCATCCTTAGACTCAGGGGCAAAGATTCTGTCTCAGTAAATCAGGC
TCTTACCGACAAATTCCTGAGTTTACCGTCAGAACTCAGAAAATCACTGACATGGGACAG
AGGAATGGAACTGGCCAGACATCTAGAATTTACTGTCAGCACCGGCGTTAAAGTTTACTT
CTGCGATCCTCAGAGTCCTTGGCAGCGGGGAACAAATGAGAACACAAATGGGCTAATTCG
GCAGTACTTTCCTAAAAAGACATGTCTTGCCCAATATACTCAACATGAACTAGATCTGGT
TGCTGCTCAGCTAAACAACAGACCGAGAAAGACACTGAAGTTCAAAACACCGAAAGAGAT
AATTGAAAGGGGTGTTGCATTGACAGATTGAATCTACAGTAGCCTTTTTAATAT
>R4
CGGTAATGACTCCAACTTATTGATAGTGTTTTATGTTCAGATAATGCCCGATGACTTTGT
CATGCAGCTCCACCGATTTTGAGAACGACAGCGACTTCCGTCCCAGCCGTGCCAGGTGCT
GCCTCAGATTCAGGTTATGCCGCTCAATTCGCTGCGTATATCGCTTGCTGATTACGTGCA
GCTTTCCCTTCAGGCGGGATTCATACAGCGGCCAGCCATCCGTCATCCATATCACCACGT
CAAAGGGTGACAGCAGGCTCATAAGACGCCCCAGCGTCGCCATAGTGCGTTCACCGAATA
CGTGCGCAACAACCGTCTTCCGGAGACTGTCATACGCGTAAAACAGCCAGCGCTGGCGCG
ATTTAGCCCCGACATAGCCCCACTGTTCGTCCATTTCCGCGCAGACGATGACGTCACTGC
CCGGCTGTATGCGCGAGGTTACCGACTGCGGCCTGAGTTTTTTAAATGACGGAAAATCGT
GTTGAGGCCAACGCCCATAATGCGGGCTGTTGCCCGGCATCCAACGCCATTCATGGCCAT
ATCAATGATTTTCTGGTGCGTACCGGGTTGAGAAGCGGTGTAAGTGAACTGCAGTTGCCA
TGTTTTACGGCAGTGAGAGCAGAGATAGCGCTGATGTCCGGCAGTGCTTTTGCCGTTACG
CACCACCCCGTCAGTAGCTGAACAGGAGGGACAGCTGATAGAAACAGAAGCCACTGGAGC
ACCTCAAAAACACCATCATACACTAAATCAGTAAGTTGGCAGCATCACCCAGAACACAAA
TGGGCTAATTCGGCAGTACTTTCCTAAAAAGACATGTCTTGCCCAATATACTCAACATGA
ACTAGATCTGGTTGCTGCTCAGCTAAACAACAGACCGAGAAAGACACTGAAGTTCAAAAC
ACCGAAAGAGATAATTGAAAGGGGTGTTGCATTGACAGATTGAATCTACAGCAGCTTTTT
TTAATAT
>R5
ATTAGACTGGCCCCCTGAATCTCCAGACAACCAATATCACTTAAATAAGTGATAGTCTTA
ATACTAGTTTTTAGACTAGTCATTGGAGAACAGATGATTGATGTCTTAGGGCCGGAGAAA
CGCAGACGGCGTACCACACAGGAAAAGATCGCAATTGTTCAGCAGAGCTTTGAACCGGGG
ATGACGGTCTCCCTCGTTGCCCGGCAACATGGTGTAGCAGCCAGCCAGTTATTTCTCTGG
CGTAAGCAATACCAGGAAGGAAGTCTTACTGCTGTCGCCGCCGGAGAACAGGTTGTTCCT
GCCTCTGAACTTGCTGCCGCCATGAAGCAGATTAAAGAACTCCAGCGCCTGCTCGGCAAG
AAAACGATGGAAAATGAACTCCTCAAAGAAGCCGTTGAATATGGACGGGCAAAAAAGTGG

ATAGCGCACGCGCCCTTATTGCCCGGGGATGGGGAGTAAGCTTAGTCAGCCGTTGTCTCC
GGGTGTCGCGTGCGCAGTTGCACGTCATTCTCAGACGAACCGATGACTGGATGGATGGCC
GCCGCAGTCGTCACACTGATGATACGGATGTGCTTCTCCGTATACACCATGTTATCGGAG
AGCTGCCAACGTATGGTTATCGTCGGGTATGGGCGCTGCTTCGCAGACAGGCAGAACTTG
ATGGTATGCCTGCGATCAATGCCAAACGTGTTTACCGGATCATGCGCCAGAATGCGCTGT
TGCTTGAGCGAAAACCTGCTGTACCGCCATCGAAACGGGCACATACAGGCAGAGTGGCCG
TGAAAGAAAGCAATCAGCGATGGTGCTCTGACGGGTTCGAGTTCTGCTGTGATAACGGAG
AGAGACTGCGTGTCACGTTCGCGCTGGACTGCTGTGATCGTGAGGCACTGCACTGGGCGG
TCACTACCGGCGGCTTCAACAGTGAAACAGTACAGGACGTCATGCTGGGAGCGGTGGAAC
GCCGCTTCGGCAACGATCTTCCGTCGTCTCCAGTGGAGTGGCTGACGGATAATGGTTCAT
GCTACCGGGCTAATGAAACACGCCAGTTCGCCCGGATGTTGGGACTTGAACCGAAGAACA
CGGCGGTGCGGAGTCCGGAGAGTAACGGAATAGCAGAGAGCTTCGTGAAAACGATAAAGC
GTGACTACATCAGTATCATGCCCAAACCAGACGGGTTAACGGCAGCAAAGAACCTTGCAG
AGGCGTTCGAGCATTATAACGAATGGCATCCGCATAGTGCGCTGGGTTATCGCTCGCCAC
GGGAATATCTGCGGCAGCGGGCTTGTAATGGGTTAAGTGATAACAGATGTCTGGAAATAT
AGGGGCAAATCCAGT
>R6
GTAATCTGATCTTACCCAGCAATAGTGGACACGCGGCTAAGTGAGTAAACTCTCAGTCAG
AGGTGACTCACATGACAAAAACAGTATCAACCAGTAAAAAACCCCGTAAACAGCATTCGC
CTGAATTTCGCAGTGAAGCCCTGAAGCTTGCTGAACGCATCGGTGTTACTGCCGCAGCCC
GTGAACTCAGCCTGTATGAATCACAACTCTACAACTGGCGCAGTAAACAGCAAAATCAGC
AGACGTCTTCTGAACGTGAACTGGAGATGTCTACCGAGATTGCACGTCTCAAACGCCAGC
TGGCAGAACGGGATGAAGAGCTGGCTATCCTCCAAAAGGCCGCGACATACTTCGCGAAGC
GCCTGAAATGAAGTATGTCTTTATTGAAAAACATCAGGCTGAGTTCAGCATCAAAGCAAT
GTGCCGCGTGCTCCGGGTGGCCCGCAGCGGCTGGTATACGTGGTGTCAGCGGCGGACAAG
GATAAGCACGCGTCAGCAGTTCCGCCAACACTGCGACAGCGTTGTCCTCGCGGCTTTTAC
CCGGTCAAAACAGCGTTACGGTGCCCCACGCCTGACGGATGAACTGCGTGCTCAGGGTTA
CCCCTTTAACGTAAAAACCGTGGCGGCAAGCCTGCGCCGTCAGGGACTGAGGGCAAAGGC
CTCCCGGAAGTTCAGCCCGGTCAGCTACCGCGCACACGGCCTGCCTGTGTCAGAAAATCT
GTTGGAGCAGGATTTTTACGCCAGTGGCCCGAACCAGAAGTGGGCAGGAGACATCACGTA
CTTACGTACAGATGAAGGCTGGCTGTATCTGGCAGTGGTCATTGACCTGTGGTCACGTGC
CGTTATTGGCTGGTCAATGTCGCCACGCATGACGGCGCAACTGGCCTGCGATGCCCTGCA
GATGGCGCTGTGGCGGCGTAAGAGGCCCCGGAACGTTATCGTTCACACGGACCGTGGAGG
CCAGTACTGTTCAGCAGATTATCAGGCGCAACTGAAGCGGCATAATCTGCGTGGAAGTAT
GAGCGCAAAAGGTTGCTGCTACGATAATGCCTGCGTGGAAAGCTTCTTTCATTCGCTGAA
AGTGGAATGTATCCATGGAGAACACTTTATCAGCCGGGAAATAATGCGGGCAACGGTGTT
TAATTATATCGAATGTGATTACAATCGGTGGCGGCGGCACAGTTGGTGTGGCGGCCTCAG
TCCGGAACAATTTGAAAACAAGAACCTCGCTTAGGCCTGTGTCCATATTACGTGGGTAGG
ATCAACCAGCCC
>R7
TATATTCACTCAGCAACCCCGGTATCAGTTCATCCAGCGCGGCTGCTTTGTTCATGGCTT
TGATGATATCCCGTTTCAGGAAATCAACATGTCGGTTTTCCAGTTCCGGAAAACGCCGCT
GCACCGACAGGGGGATCCCGTCGAGAATACTGGCAATTTCACCTGCGATCCGCGACAGCA
CGAAAGTACAGAATGCGGTTTCCACCACTTCAGCGGAGTCTCTGGCATTTTTCAGCTCCT
GTGCATCGGCCTGCGCACGCGTAAGTCGATGGCGTTCGTACTCAATAGTCCCTGGCTGGA
GATCTGTCTCGCTGGCCTGCAGCAGTTCTTCAACCTCCCGGCGCAGCTTTTCGTTCTCAA
TTTCAGCATCCCTTTCGGCATACCATCTTATAACGGCGGCAGAGTCATAAAGCACCTCAT
TACCCTTGCCACCGCCTCGCAGAACGGGCATTCCCTGTTCCTGCCAGTTCTGAATGGTAC

GGATACTCGCACCGAAAATGTCAGCCAGCTGCTTTTTGTTGACTTCCATTGCACATTCCA
CGGACAAAAACAGAGAAAGGAAACGACAGAGGCCAAAAAGCTCGCTTTCAGCACCTGTCG
TTTCCTTTCTTTTCAGGGGGTATTTTAAATAAAAACATTAAGTTACGACGAAGAAGAACG
GAAACACCTTAAACCGGAAAATTTTCATAAATAGCGAAAACCCGCGAGGTCGCCGCCCCG
TAACCTGTCGGATCACCGGAAAGGACCCACAAAATGATAATAATTATCATCTACATATCA
CAACGTGCATCGACGCCATCAAACCACGTCAAATAATCAATTATGACGCAGGTATCGTAT
TAATTGATCTGCATCAACTTAACGTAAAAACAACTTCAGACAATACAAATCAGCGACACT
GAATACGGGGCAACCTCATGTCAACGAAGAACAGAACCCGCAGAACAACAACCCGCAACA
TCCGCTTTCCTAACCAAATGATTGAACAAATTAACATCGCTCTTGAGCAAAAAGGGTCCG
GGAATTTCTCAGCCTGGGTCATTGAAGCCTGCCGCCGGAGGCTAACGTCAGAAAAGAGAG
>R8
AACGCAAAATTGCCTGATGCGCTACGCTTATCAGGCCTACGCAATCTCTGCAATATATTG
AATTTGCGTGCTTTTGTAGGCCGGATAAGGCGTTCACGCCGCATCCGGCA
>R9
TCATCTTGTTGCGCTCCTTTCATGAGCTAAGCAACATATTGCCACTGGCGCAAGGAGCGC
GCAGGGGGCGGCCAATCGCCGCCGCCCCCTGCACCCCCGGGCTCTGGCGAACAAAATCGC
CGCTGCGCGGTGCCCTCGACTTATCCCTTACGGCTACCGGGTCGGGCGCGAGGTAACATC
CCTGTAAAACGCGCCCTCAGCCCACATCCATGTGGGCTGCCCCGGCCTTCAGGGAACGCC
TCGGCAATTTTGACGCCACCAA
>R10
CTTCTCCCAAACCAAGGTTTTCGAGAGCCGTTTGCACAGTGCCATCCAATTTGATATCGC
CAAACGGATTCTTGCGGCTTAACAGCAGCGCACGAAGCGCGGTAAGCAACTGGTCATGCC
GCCCCTTCTCCAGGCTGGCACCGGAGGCCTCCACAACGCTGCAAAGCTCCTCCTGCAACA
TGTCAAAGTAGTCATCATCCAGATCGGTGGCAGGCGTGCCGGTCTGGGGGTTACCACGGG
TA
>R11
TCGTTGATGGTCAGAACGACCAGTTCACACCAGCCACCGCGTTCCACGGGGATTCCACAC
CGGCACCATGGCTATACCCCACCCCAAGATGCCCGCTAAACGTACTGCTGAATGAGGCTT
TAATACCTGCCTGGTATATTCCACGTCTGCCCGACAAATAATTGACGAAATTACCGTCAC
TATTCACCTTCACCCGGTTATCATCGACAAATTCTTTGCGCACAGCCGCCTTCAGCCACG
GCTCAACTTCCATACCGTTCCCCAGACGCATGTTGTAACTCAGCGTTGCACCCAGTTCAC
GATATATACTGCGGGTATCGACTGATTTCGATTCCATGCCATTGGATAAATGATA
>R12
TGGTGGTGGGGGAAGGATTCGAACCTTCGAAGTCGATGACGGCAGATTTACAGTCTGCTC
CCTTTGGCCGCTCGGGAACCCCACC
>R13
CCAACCGCAGCACGTTCTTGCATAAGACGTGACTGCGGTTTTTCAACTATTCAGATACAT
CACTCCCATCACATTCATTCCTCCGCATCAAAGGCATATAGGCTATATCACCTTGATATT
TTTCTTCTTCAGATAAAAACTGTTATCTATGTATACTTTTAAACCCAATCCGTGTAGAGT
CTCTACATAAGATAGTTTGCAGTTGCCACTTCAGCTTGCGCCATAAACCGCCTGATTTTT
GCCGCCACCTGTTAGCATTCCTGTATACCTGAAACGACAATGTTTATCTACGAACTTTAA
GAACACCCAAGATAAAAATTGTCAACTATATCATATATAACACATTACTAATTCGAGGCT
ATATGAACAGCATACTGATAATCACATCGCTCCTTATCATATTCAGCATTTTTAGTCATG
CCCTAATAAAATTAGGGATTGGCATATCCAATAACCCAGACAAAACCGATGTATAAGTCA
ACATATCCTGAATCAGACATACAATATCGCAATGAAAATCAATAATATTTTAAGGAATAT
CTTCATGAAATCAAAAGACACCCTAAAGTGGTTCCCTGCGCAGCTTCCTGAAGTAAGAAT
TATCCTAGGGGATGCTGTAGTGGAAGTAGCAAAACAGGGAAGACCTATCAATACCAGAAC
ATTGCTTGATTACATTGAAGGAAACATAAAGAAAAAATCATGGCTGGATAACAAAGAATT

ATTACAAACAGCGATATCAGTTCTTAAAGACAACCAAAATTTAAATGGTAAAATGTAATA
TAATAAACTTACTTTTTTATCATTTTTCCACTTTAACAACATTTTGCTCCACTTTTCCAC
GACCAAACAACTTGAAATCTGGTTAAAATAACACGCAACACTATTCTTCTTCCTTGAGTC
CGCCCGGAACTCGAAAAACAAACCGAGTTAAAGCCATTTTTCACAAAATCGATTTTGGGT
CTCACCAAAATTACGGGGTTGCATACGCATTCGTTTATTTTCGAACGTGTACATACAAAT
ATGCACAAAAATAATCATAATTATTTTCTGAGATGCATTATGATATGAACACCAATTTCG
TATAGAGTCTCACTATGTCTCAAATTTTTGCTTACTGTCGGATATCAACGCTGGATCAGA
CCACCGAAAATCAACGCCGGGAAATCGAAAGTGCAGGTTTTAAAATCAAACCTCAGCAAA
TAATCGAAGAACACATTAGCGGCTCAGCAGCAACCAGTGAGCGTCCTGGTTTTAACCGGT
TGCTTGCTCGCCTGAAATGTGGTGATCAATTGATTGTGACAAAACTGGATCGCCTTGGTT
GTAATGCAATGGATATCAGGAAAACAGTGGAACAACTGACCGAAACAGGTATCAGAGTGC
ATTGCTTAGCATTGGGGGGCATTGACCTGACCAGTCCAACAGGAAAAATGATGATGCAAG
TAATTTCAGCAGTCGCTGAATTTGAACGAGACCTTTTACTTGAACGCACTCATTCCGGGA
TAGTAAGAGCCCGCGGCGCAGGGAAACGTTTTGGTCGACCACCTGTGTTAAATGAAGAAC
AGAAACAGGCGGTATTCGAACGAATTAAGTCAGGTGTAAGTATAAGTGCCATTGCCCGGG
AATTCAAAACCTCGCGGCAAACCATTTTAAGAGCCAAAGCAAAACTTCAGACACCTGACA
TATAAAAAATAATCTCGGTGTGAGATGCTTTACGTCTTCCAAGCCCCCTTCCTTGCCGTA
AATGGAAAGATACATCTAATTATAGAATTTATATGTTTTACCCTACGGCAGTGCTGGCCA
TTCAATATCCTGTGCAGTTGACGTATCAACACGGTTCAGCAATACCCGATACTTTTTCCA
TGCTTCCAGCAACGAGATTTCTTCCTCCGTTGCAATTTCCAGATCTGCAGCATCCTGAAG
CGGCGCAATATGCTCACTGGCTACCTGCATCAGGTTGTTTTTTGTTTCTTCCGCCTCCCG
GATCCGGAACAGTTTTTCTGCTTCCGTATCCTTCACCCAGGCTGTGCCGTTCCACTTCTG
AAACTCCCCTTCCGGCGATAACCAGGTAACATTTTCCGGTAACGGACCGAGTTCAGAAAT
AAATAACGCGTCGCCGGAAGCCACGTCATAAACCGTTTTACCCCGATGATCTTCAACGAG
ATGCCACGATGACTCATCACTGTTGAAAACAGCCACGAAGCCAGCCGGAATATCTGGCGG
TGCAATATCGGTACTGTTTGCTGGCAGACCTGTATGAGGCGGAATATATGCATCACCTTC
ACCAATAAATTCATTAGTTCCGGCCAGCAGATTATAAATTTTTATGGTCCGTGGTTGTTC
ACTCATTCTGAATGCCATTATGCAAGCCTCACAATATAGTTAAATGCGATGTTTTTGACG
GTGTTTTCCGCGTTACCAGCAGCGTTAACGGTGATGGTGTGTCCATGTGAACCAATCGCA
ACGGAGTGCGTATGAGCACCAATACCGACAGTATGCGCATGCGCACCTGCGCTTGCAGCA
GTGCCGGACAGCGAGTGGGTATGTGCGCCAGCAGATGATGT
>R14
GCGGGAATAGCTCAGTTGGTAGAGCACGACCTTGCCAAGGTCGGGGTCGCGAGTTCGAGT
CTCGTTTCCCGCTCCA
>R15
TAATATTCGCCCCGTTCACACGATTCCTCTGTAGTTCAGTCGGTAGAACGGCGGACTGTT
AATCCGTATGTCACTGGTTCGAGTCCAGTCAGAGGAGCCAAATT
>R16
CGCGGGGTGGAGCAGCCTGGTAGCTCGTCGGGCTCATAACCCGAAGGTCGTCGGTTCAAA
TCCGGCCCCCGCAACCA
>R17
AGTTAGGGAAGGTGCGAATAAGCGGGGAAATTCTTCTCGGCTGACTCAGTCATTTCATTT
CTTCATGTTTGAGCCGATTTTTTCTCCCGTAAATGCCTTGAATCAGCCTATTTAGACCGT
TTCTTCGCCATTTAAGGCGTTATCCCCAGTTTTTAGTGAGATCTCTCCCACTGACGTATC
ATTTGGTCCGCCCGAAACAGGTTGGCCAGCGTGAATAACATCGCCAGTTGGTTATCGTTT
TTCAGCAACCCCTTGTATCTGGCTTTCACGAAGCCGAACTGTCGCTTGATGATGCGAAAT
GGGTGCTCCACCCTGGCCCGGATGCTGGCTTTCATGTATTCGATGTTGATGGCCGTTTTG
TTCTTGCGTGGATGCTGTTTCAAGGTTCTTACCTTGCCGGGGCGCTCGGCGATCAGCCAG

TCCACATCCACCTCGGCCAGCTCCTCGCGCTGTGGCGCCCCTTGGTAGCCGGCATCGGCT
GAGACAAATTGCTCCTCTCCATGCAGCAGATTACCCAGCTGATTGAGGTCATGCTCGTTG
GCCGCGGTGGTGACCAGGCTGTGGGTCAGGCCACTCTTGGCATCGACACCAATGTGGGCC
TTCATGCCAAAGTGCCACTGATTGCCTTTCTTGGTCTGATGCATCTCCGGATCGCGTTGC
TGCTCTTTGTTCTTGGTCGAGCTGGGTGCCTCAATGATGGTGGCATCGACCAAGGTGCCT
TGAGTCATCATGACGCCTGCTTCGGCCAGCCAGCGATTGATGGTCTTGAACAATTGGCGG
GCCAGTTGATGCTGCTCCAGCAGGTGGCGGAAATTCATGATGGTGGTGCGGTCCGGCAAG
GCGCTATCCAGGGATAACCGGGCAAACAGACGCATGGAGGCGATTTCGTACAGAGCATCT
TCCATCGCGCCATCGCTCAGGTTGTACCAATGCTGCATGCAGTGAATGCGTAGCATGGTT
TCCAGCGGATAAGGTCGCCGGCCATTACCAGCCTTGGGGTAAAACGGCTCGATGACTTCC
ACCATGTTTTGCCATGGCAGAATCTGCTCCATGCGGGACAAGAAAATCTCTTTTCTGGTC
TGACGGCGCTTACTGCTGAATTCACTGTCGGCGAAGGTAAGTTGATGACTCATGATGAAC
CCTGTTCTATGGCTCCAGATGACAAACATGATCTCATATCAGGGACTTGTTCGCACCTTC
CTTAGT
>R18
GAAAAGCAAAAAGGCCATCCGTCAGGATGGCCTTCCGCTTAATTTGATGCCTGGCAGTTT
ATGGCGGGCGTCCTGCCCGCCACCCTCCGGGCCGTTGCTTCGCAACGTTCAAATCCGCTC
CCGGCGGATTTGTCCTACTCAGGAGAGCGTTCACCGACAAACAACAGATAAAACAAAAGG
CCCAGTCTTCCGACTGAGCCTTTCGTTTTATTTGATGCCTGGCAGTTCCCTACTCTCGCA
TGGGGAGACCCCACACTACCATCGGCGCTACGGCGTTTCACTTCTGAGTTCGGCATGGGG
TCAGGTGGGACCACCGCGCTACTGCCGCCAGGCAAATTCTGTTTTATCAGACCGCTTCTG
CGTTCTGATTTAATCTGTATCAGGCTGAAAATCTTCTCTCATCCGCCAAAACAGCTTCGG
CGTTGTAAGGTTAAGCCTCACGGTTCATTAGTACCGGTTAGCTCAACGCATCGCTGCGCT
TACACACCCGGCCTATCAACGTCGTCGTCTTCAACGTTCCTTCAGGACCCTTAAAGGGTC
AGGGAGAACTCATCTCGGGGCAAGTTTCGTGCTTAGATGCTTTCAGCACTTATCTCTTCC
GCATTTAGCTACCGGGCAGTGCCATTGGCATGACAACCCGAACACCAGTGATGCGTCCAC
TCCGGTCCTCTCGTACTAGGAGCAGCCCCCCTCAGTTCTCCAGCGCCCACGGCAGATAGG
GACCGAACTGTCTCACGACGTTCTAAACCCAGCTCGCGTACCACTTTAAATGGCGAACAG
CCATACCCTTGGGACCTACTTCAGCCCCAGGATGTGATGAGCCGACATCGAGGTGCCAAA
CACCGCCGTCGATATGAACTCTTGGGCGGTATCAGCCTGTTATCCCCGGAGTACCTTTTA
TCCGTTGAGCGATGGCCCTTCCATTCAGAACCACCGGATCACTATGACCTGCTTTCGCAC
CTGCTCGCGCCGTCACGCTCGCAGTCAAGCTGGCTTATGCCATTGCACTAACCTCCTGAT
GTCCGACCAGGATTAGCCAACCTTCGTGCTCCTCCGTTACTCTTTAGGAGGAGACCGCCC
CAGTCAAACTACCCACCAGACACTGTCCGCAACCCGGATTACGGGTCAACGTTAGAACAT
CAAACATTAAAGGGTGGTATTTCAAGGTCGGCTCCATGCAGACTGGCGTCCACACTTCAA
AGCCTCCCACCTATCCTACACATCAAGGCTCAATGTTCAGTGTCAAGCTATAGTAAAGGT
TCACGGGGTCTTTCCGTCTTGCCGCGGGTACACTGCATCTTCACAGCGAGTTCAATTTCA
CTGAGTCTCGGGTGGAGACAGCCTGGCCATCATTACGCCATTCGTGCAGGTCGGAACTTA
CCCGACAAGGAATTTCGCTACCTTAGGACCGTTATAGTTACGGCCGCCGTTTACCGGGGC
TTCGATCAAGAGCTTCGCTTGCGCTAACCCCATCAATTAACCTTCCGGCACCGGGCAGGC
GTCACACCGTATACGTCCACTTTCGTGTTTGCACAGTGCTGTGTTTTTAATAAACAGTTG
CAGCCAGCTGGTATCTTCGACTGATTTCAGCTCCATCCGCGAGGGACCTCACCTACATAT
CAGCGTGCCTTCTCCCGAAGTTACGGCACCATTTTGCCTAGTTCCTTCACCCGAGTTCTC
TCAAGCGCCTTGGTATTCTCTACCTGACCACCTGTGTCGGTTTGGGGTACGATTTGATGT
TACCTGATGCTTAGAGGCTTTTCCTGGAAGCAGGGCATTTGTTGCTTCAGCACCGTAGTG
CCTCGTCATCACGCCTCAGCCTTGATTTTCCGGATTTGCCTGGAAAACCAGCCTACACGC
TTAAACCGGGACAACCGTCGCCCGGCCAACATAGCCTTCTCCGTCCCCCCTTCGCAGTAA
CACCAAGTACAGGAATATTAACCTGTTTCCCATCGACTACGCCTTTCGGCCTCGCCTTAG

GGGTCGACTCACCCTGCCCCGATTAACGTTGGACAGGAACCCTTGGTCTTCCGGCGAGCG
GGCTTTTCACCCGCTTTATCGTTACTTATGTCAGCATTCGCACTTCTGATACCTCCAGCA
TGCCTCACAGCACACCTTCGCAGGCTTACAGAACGCTCCCCTACCCAACAACGCATAAGC
GTCGCTGCCGCAGCTTCGGTGCATGGTTTAGCCCCGTTACATCTTCCGCGCAGGCCGACT
CGACCAGTGAGCTATTACGCTTTCTTTAAATGATGGCTGCTTCTAAGCCAACATCCTGGC
TGTCTGGGCCTTCCCACATCGTTTCCCACTTAACCATGACTTTGGGACCTTAGCTGGCGG
TCTGGGTTGTTTCCCTCTTCACGACGGACGTTAGCACCCGCCGTGTGTCTCCCGTGATAA
CATTCTCCGGTATTCGCAGTTTGCATCGGGTTGGTAAGTCGGGATGACCCCCTTGCCGAA
ACAGTGCTCTACCCCCGGAGATGAATTCACGAGGCGCTACCTAAATAGCTTTCGGGGAGA
ACCAGCTATCTCCCGGTTTGATTGGCCTTTCACCCCCAGCCACAAGTCATCCGCTAATTT
TTCAACATTAGTCGGTTCGGTCCTCCAGTTAGTGTTACCCAACCTTCAACCTGCCCATGG
CTAGATCACCGGGTTTCGGGTCTATACCCTGCAACTTAACGCCCAGTTAAGACTCGGTTT
CCCTTCGGCTCCCCTATTCGGTTAACCTTGCTACAGAATATAAGTCGCTGACCCATTATA
CAAAAGGTACGCAGTCACACGCCTAAGCGTGCTCCCACTGCTTGTACGTACACGGTTTCA
GGTTCTTTTTCACTCCCCTCGCCGGGGTTCTTTTCGCCTTTCCCTCACGGTACTGGTTCA
CTATCGGTCAGTCAGGAGTATTTAGCCTTGGAGGATGGTCCCCCCATATTCAGACAGGAT
ACCACGTGTCCCGCCCTACTCATCGAGCTCACAGCATGTGCATTTTTGTGTACGGGGCTG
TCACCCTGTATCGCGCGCCTTTCCAGACGCTTCCACTAACACACACACTGATTCAGGCTC
TGGGCTGCTCCCCGTTCGCTCGCCGCTACTGGGGGAATCTCGGTTGATTTCTTTTCCTCG
GGGTACTTAGATGTTTCAGTTCCCCCGGTTCGCCTCATTAACCTATGGATTCAGTTAATG
ATAGTGTGTCGAAACACACTGGGTTTCCCCATTCGGAAATCGCCGGTTATAACGGTTCAT
ATCACCTTACCGACGCTTATCGCAGATTAGCACGTCCTTCATCGCCTCTGACTGCCAGGG
CATCCACCGTGTACGCTTAGTCGCTTAACCTCACAACCCGAAGATGTTTCACTCGATTCA
TCATCGAGTTGCGAAAATTTGAGAGACTCACGAACAACTCTCATTGTTCAGTGTTTCAAT
TTTCAGCTTGATCCAGATTTTTAAAGAGCAAATATATCAAACAACACCCAAAAATCAGTT
TTGAGATATTAAGGTCGGCGACTTTCACTCACAAACCAGCAAGTGGCGTCCCCTAGGGGA
TTCGAACCCCTGTTACCGCCGTGAAAGGGCGGTGTCCTGGGCCTCTAGACGAAGGGGACA
CGAAAATTGCTTATCACGCGTTGCGTGATATTTTCGTGTAGGGTGAGCTTTCATTAATAG
AAAGCGAACGGCCTTATTCTCTTCAGCCTCACTCCCAACGCGTAAACGCCTTGCTATTCA
CTTTCCATCAGACAATCTGTGTGAGCACTACAAAGTACGCTTCTTTAAGGTAAGGAGGTG
ATCCAACCGCAGGTTCCCCTACGGTTACCTTGTTACGACTTCACCCCAGTCATGAATCAC
AAAGTGGTAAGCGCCCTCCCGAAGGTTAAGCTACCTACTTCTTTTGCAACCCACTCCCAT
GGTGTGACGGGCGGTGTGTACAAGGCCCGGGAACGTATTCACCGTGGCATTCTGATCCAC
GATTACTAGCGATTCCGACTTCATGGAGTCGAGTTGCAGACTCCAATCCGGACTACGACG
CACTTTATGAGGTCCGCTTGCTCTCGCGAGGTCGCTTCTCTTTGTATGCGCCATTGTAGC
ACGTGTGTAGCCCTGGTCGTAAGGGCCATGATGACTTGACGTCATCCCCACCTTCCTCCA
GTTTATCACTGGCAGTCTCCTTTGAGTTCCCGGCCGGACCGCTGGCAACAAAGGATAAGG
GTTGCGCTCGTTGCGGGACTTAACCCAACATTTCACAACACGAGCTGACGACAGCCATGC
AGCACCTGTCTCACGGTTCCCGAAGGCACATTCTCATCTCTGAAAACTTCCGTGGATGTC
AAGACCAGGTAAGGTTCTTCGCGTTGCATCGAATTAAACCACATGCTCCACCGCTTGTGC
GGGCCCCCGTCAATTCATTTGAGTTTTAACCTTGCGGCCGTACTCCCCAGGCGGTCGACT
TAACGCGTTAGCTCCGGAAGCCACGCCTCAAGGGCACAACCTCCAAGTCGACATCGTTTA
CGGCGTGGACTACCAGGGTATCTAATCCTGTTTGCTCCCCACGCTTTCGCACCTGAGCGT
CAGTCTTCGTCCAGGGGGCCGCCTTCGCCACCGGTATTCCTCCAGATCTCTACGCATTTC
ACCGCTACACCTGGAATTCTACCCCCCTCTACGAGACTCAAGCTTGCCAGTATCAGATGC
AGTTCCCAGGTTGAGCCCGGGGATTTCACATCTGACTTAACAAACCGCCTGCGTGCGCTT
TACGCCCAGTAATTCCGATTAACGCTTGCACCCTCCGTATTACCGCGGCTGCTGGCACGG
AGTTAGCCGGTGCTTCTTCTGCGGGTAACGTCAATGAGCAAAGGTATTAACTTTACTCCC

TTCCTCCCCGCTGAAAGTACTTTACAACCCGAAGGCCTTCTTCATACACGCGGCATGGCT
GCATCAGGCTTGCGCCCATTGTGCAATATTCCCCACTGCTGCCTCCCGTAGGAGTCTGGA
CCGTGTCTCAGTTCCAGTGTGGCTGGTCATCCTCTCAGACCAGCTAGGGATCGTCGCCTA
GGTGAGCCGTTACCCCACCAACAAGCTAATCCCATCTGGGCACATCCGATGGCAAGAGGC
CCGAAGGTCCCCCTCTTTGGTCTTGCGACATTATGCGGTATTAGCTACCGTTTCCAGTAG
TTATCCCCCTCCATCAGGCAGCTTCCCAGACATTACTCACCCGTCCGCCACTCGTCAGCA
AAGAAGCAAGCTGCTTCCTGTTACCGTTCGACTTGCATGTGTTAGGCCTGCCGCCAGCGT
TCAATCTGAGCCATGATCAAACTCTTCAATTTAAAAGTTTGACGCTCAAAGAATTAAACT
TCGTAATGAATTACGTGTTCACTCTTGAGACTTGGTATTCATTTTTCGTCTTGCGACGTT
AAGAATCCGTATCTTCGAGTGCCCACACAGATTGTCTGATAAATTGTTAAAGAGCAGTGC
CGCTTCGCTTTTTCTCAGCGGCGCGGGGTGTGCATAATACGCCTTCCCGCTACAGAGTCA
AGCATTTATTTGCGCTTTCTCTGCCGAAATTCTCAGGAGAACCCCGCCGACCCGGCGGCG
TGTTTGCCGTTGTTCCGTGTCAGTGGTGGCGCATTATAGGGAGTTATTCCGGCCTGACAA
G
>R19
CTTGTCAGGCCGGAATAACTCCCTATAATGCGCCACCACTGACACGGAACAACGGCAAAC
ACGCCGCCGGGTCAGCGGGGTTCTCCTGAGAACTCCGGCAGAGAAAGCAAAAATAAATGC
TTGACTCTGTAGCGGGAAAGCGTATTATGCACACCCCGCGCCGCTGAGAAAAAGCAAAGC
GGCACTGCTCTTTAACAATTTATCAGACAATCTGTGTGGGCACTCGAAGATACGGATTCT
TAACGTCGCAAGACGAAAAATGAATACCAAGTCTCAAGAGTGAACACGTAATTCATTACG
AAGTTTAATTCTTTGAGCATCAAACTTTTAAATTGAAGAGTTTGATCATGGCTCAGATTG
AACGCTGGCGGCAGGCCTAACACATGCAAGTCGAACGGTAACAGGAAACAGCTTGCTGTT
TCGCTGACGAGTGGCGGACGGGTGAGTAATGTCTGGGAAACTGCCTGATGGAGGGGGATA
ACTACTGGAAACGGTAGCTAATACCGCATAACGTCGCAAGACCAAAGAGGGGGACCTTCG
GGCCTCTTGCCATCAGATGTGCCCAGATGGGATTAGCTAGTAGGTGGGGTAACGGCTCAC
CTAGGCGACGATCCCTAGCTGGTCTGAGAGGATGACCAGCCACACTGGAACTGAGACACG
GTCCAGACTCCTACGGGAGGCAGCAGTGGGGAATATTGCACAATGGGCGCAAGCCTGATG
CAGCCATGCCGCGTGTATGAAGAAGGCCTTCGGGTTGTAAAGTACTTTCAGCGGGGAGGA
AGGGAGTAAAGTTAATACCTTTGCTCATTGACGTTACCCGCAGAAGAAGCACCGGCTAAC
TCCGTGCCAGCAGCCGCGGTAATACGGAGGGTGCAAGCGTTAATCGGAATTACTGGGCGT
AAAGCGCACGCAGGCGGTTTGTTAAGTCAGATGTGAAATCCCCGGGCTCAACCTGGGAAC
TGCATCTGATACTGGCAAGCTTGAGTCTCGTAGAGGGGGGTAGAATTCCAGGTGTAGCGG
TGAAATGCGTAGAGATCTGGAGGAATACCGGTGGCGAAGGCGGCCCCCTGGACGAAGACT
GACGCTCAGGTGCGAAAGCGTGGGGAGCAAACAGGATTAGATACCCTGGTAGTCCACGCC
GTAAACGATGTCGACTTGGAGGTTGTGCCCTTGAGGCGTGGCTTCCGGAGCTAACGCGTT
AAGTCGACCGCCTGGGGAGTACGGCCGCAAGGTTAAAACTCAAATGAATTGACGGGGGCC
CGCACAAGCGGTGGAGCATGTGGTTTAATTCGATGCAACGCGAAGAACCTTACCTGGTCT
TGACATCCACGGAAGTTTTCAGAGATGAGAATGTGCCTTCGGGAACCGTGAGACAGGTGC
TGCATGGCTGTCGTCAGCTCGTGTTGTGAAATGTTGGGTTAAGTCCCGCAACGAGCGCAA
CCCTTATCCTTTGTTGCCAGCGGTCCGGCCGGGAACTCAAAGGAGACTGCCAGTGATAAA
CTGGAGGAAGGTGGGGATGACGTCAAGTCATCATGGCCCTTACGACCAGGGCTACACACG
TGCTACAATGGCGCATACAAAGAGAAGCGACCTCGCGAGAGCAAGCGGACCTCATAAAGT
GCGTCGTAGTCCGGATTGGAGTCTGCAACTCGACTCCATGAAGTCGGAATCGCTAGTAAT
CGTGGATCAGAATGCCACGGTGAATACGTTCCCGGGCCTTGTACACACCGCCCGTCACAC
CATGGGAGTGGGTTGCAAAAGAAGTAGGTAGCTTAACCTTCGGGAGGGCGCTTACCACTT
TGTGATTCATGACTGGGGTGAAGTCGTAACAAGGTAACCGTAGGGGAACCTGCGGTTGGA
TCACCTCCTTACCTTAAAGAAGCGTTCTTTGCAGTGCTCACACAGATTGTCTGATAGGAA
GTGAAAAGCAAGGCGTCTTGCGAAGCAGACTGATACGTCCCCTTCGTCTAGAGGCCCAGG

ACACCGCCCTTTCACGGCGGTAACAGGGGTTCGAATCCCCTAGGGGACGCCACTTGCTGG
TTTGTGAGTGAAAGTCACCTGCCTTAATATCTCAAAACTCATCTTCGGGTGATGTTTGAG
ATATTTGCTCTTTAAAAATCTGGATCAAGCTGAAAATTGAAACACTGAACAACGAAAGTT
GTTCGTGAGTCTCTCAAATTTTCGCAACACGATGATGAATCGAAAGAAACATCTTCGGGT
TGTGAGGTTAAGCGACTAAGCGTACACGGTGGATGCCCTGGCAGTCAGAGGCGATGAAGG
ACGTGCTAATCTGCGATAAGCGTCGGTAAGGTGATATGAACCGTTATAACCGGCGATTTC
CGAATGGGGAAACCCAGTGTGTTTCGACACACTATCATTAACTGAATCCATAGGTTAATG
AGGCGAACCGGGGGAACTGAAACATCTAAGTACCCCGAGGAAAAGAAATCAACCGAGATT
CCCCCAGTAGCGGCGAGCGAACGGGGAGCAGCCCAGAGCCTGAATCAGTATGTGTGTTAG
TGGAAGCGTCTGGAAAGGCGCGCGATACAGGGTGACAGCCCCGTACACAAAAATGCACAT
ATTGTGAGCTCGATGAGTAGGGCGGGACACGTGGTATCCTGTCTGAATATGGGGGGACCA
TCCTCCAAGGCTAAATACTCCTGACTGACCGATAGTGAACCAGTACCGTGAGGGAAAGGC
GAAAAGAACCCCGGCGAGGGGAGTGAAAAAGAACCTGAAACCGTGTACGTACAAGCAGTG
GGAGCACGCTTAGGCGTGTGACTGCGTACCTTTTGTATAATGGGTCAGCGACTTATATTC
TGTAGCAAGGTTAACCGAATAGGGGAGCCGAAGGGAAACCGAGTCTTAACTGGGCGTTAA
GTTGCAGGGTATAGACCCGAAACCCGGTGATCTAGCCATGGGCAGGTTGAAGGTTGGGTA
ACACTAACTGGAGGACCGAACCGACTAATGTTGAAAAATTAGCGGATGACTTGTGGCTGG
GGGTGAAAGGCCAATCAAACCGGGAGATAGCTGGTTCTCCCCGAAAGCTATTTAGGTAGC
GCCTCGTGAATTCATCTCCGGGGGTAGAGCACTGTTTCGGCAAGGGGGTCATCCCGACTT
ACCAACCCGATGCAAACTGCGAATACCGGAGAATGTTATCACGGGAGACACACGGCGGGT
GCTAACGTCCGTCGTGAAGAGGGAAACAACCCAGACCGCCAGCTAAGGTCCCAAAGTCAT
GGTTAAGTGGGAAACGATGTGGGAAGGCCCAGACAGCCAGGATGTTGGCTTAGAAGCAGC
CATCATTTAAAGAAAGCGTAATAGCTCACTGGTCGAGTCGGCCTGCGCGGAAGATGTAAC
GGGGCTAAACCATGCACCGAAGCTGCGGCAGCGACGCTTATGCGTTGTTGGGTAGGGGAG
CGTTCTGTAAGCCTGCGAAGGTGTGCTGTGAGGCATGCTGGAGGTATCAGAAGTGCGAAT
GCTGACATAAGTAACGATAAAGCGGGTGAAAAGCCCGCTCGCCGGAAGACCAAGGGTTCC
TGTCCAACGTTAATCGGGGCAGGGTGAGTCGACCCCTAAGGCGAGGCCGAAAGGCGTAGT
CGATGGGAAACAGGTTAATATTCCTGTACTTGGTGTTACTGCGAAGGGGGGACGGAGAAG
GCTATGTTGGCCGGGCGACGGTTGTCCCGGTTTAAGCGTGTAGGCTGGTTTTCCAGGCAA
ATCCGGAAAATCAAGGCTGAGGCGTGATGACGAGGCACTACGGTGCTGAAGCAACAAATG
CCCTGCTTCCAGGAAAAGCCTCTAAGCATCAGGTAACATCAAATCGTACCCCAAACCGAC
ACAGGTGGTCAGGTAGAGAATACCAAGGCGCTTGAGAGAACTCGGGTGAAGGAACTAGGC
AAAATGGTGCCGTAACTTCGGGAGAAGGCACGCTGATATGTAGGTGAAGCGACTTGCTCG
TGGAGCTGAAATCAGTCGAAGATACCAGCTGGCTGCAACTGTTTATTAAAAACACAGCAC
TGTGCAAACACGAAAGTGGACGTATACGGTGTGACGCCTGCCCGGTGCCGGAAGGTTAAT
TGATGGGGTCAGCGCAAGCGAAGCTCTTGATCGAAGCCCCGGTAAACGGCGGCCGTAACT
ATAACGGTCCTAAGGTAGCGAAATTCCTTGTCGGGTAAGTTCCGACCTGCACGAATGGCG
TAATGATGGCCAGGCTGTCTCCACCCGAGACTCAGTGAAATTGAACTCGCTGTGAAGATG
CAGTGTACCCGCGGCAAGACGGAAAGACCCCGTGAACCTTTACTATAGCTTGACACTGAA
CATTGAGCCTTGATGTGTAGGATAGGTGGGAGGCTTTGAAGTGTGGACGCCAGTCTGCAT
GGAGCCGACCTTGAAATACCACCCTTTAATGTTTGATGTTCTAACGTGGACCCGTGATCC
GGGTTGCGGACAGTGTCTGGTGGGTAGTTTGACTGGGGCGGTCTCCTCCTAAAGAGTAAC
GGAGGAGCACGAAGGTTGGCTAATCCTGGTCGGACATCAGGAGGTTAGTGCAATGGCATA
AGCCAGCTTGACTGCGAGCGTGACGGCGCGAGCAGGTGCGAAAGCAGGTCATAGTGATCC
GGTGGTTCTGAATGGAAGGGCCATCGCTCAACGGATAAAAGGTACTCCGGGGATAACAGG
CTGATACCGCCCAAGAGTTCATATCGACGGCGGTGTTTGGCACCTCGATGTCGGCTCATC
ACATCCTGGAGCTGAAGTAGGTCCCAAGGGTATGGCTGTTCGCCATTTAAAGTGGTACGC
GAGCTGGGTTTAGAACGTCGTGAGACAGTTCGGTCCCTATCTGCCGTGGGCGCTGGAGAA

CTGAGGGGGGCTGCTCCTAGTACGAGAGGACCGGAGTGGACGCATCACTGGTGTTCGGGT
TGTCATGCCAATGGCACTGCCCGGTAGCTAAATGCGGAAGAGATAAGTGCTGAAAGCATC
TAAGCACGAAACTTGCCCCGAGATGAGTTCTCCCTGACTCCTTGAGAGTCCTGAAGGAAC
GTTGAAGACGACGACGTTGATAGGCCGGGTGTGTAAGCGCAGCGATGCGTTGAGCTAACC
GGTACTAATGAACCGTGAGGCTTAACCTTACAACGCCGAAGATGTTTTGGCGGATGAGAG
AAGATTTTCAGCCTGATACAGATTAAATCAGAACGCAGAAGCGGTCTGATGAAACAGAAT
TTGCCTGGCGGCCGTAGCGCGGTGGTCCCACCTGACCCCATGCCGAACTCAGAAGTGAAA
CGCCGTAGCGCCGATGGTAGTGTGGGGTCTCCCCATGCGAGAGTAGGGAACTGCCAGGCA
TCAAATTAAGC
>R20
TGGTGCGAGGGGGGGGACTTGAACCCCCACGTCCGTAAGGACACTAACACCTGAAGCTAG
CGCGTCTACCAATTCCGCCACCTTCGCAT
>R21
CTTGTCAGGCCGGAATAACTCCCTATAATGCGCCACCACTGACACGGAACAACGGCAAAC
ACGCCGCCGGGTCAGCGGGGTTCTCCTGAGAACTCCGGCAGAGAAAGCAAAAATAAATGC
TTGACTCTGTAGCGGGAAGGCGTATTATGCACACCCCGCGCCGCTGAGAAAAAGCGAAGC
GGCACTGCTCTTTAACAATTTATCAGACAATCTGTGTGGGCACTCGAAGATACGGATTCT
TAACGTCGCAAGACGAAAAATGAATACCAAGTCTCAAGAGTGAACACGTAATTCATTACG
AAGTTTAATTCTTTGAGCGTCAAACTTTTAAATTGAAGAGTTTGATCATGGCTCAGATTG
AACGCTGGCGGCAGGCCTAACACATGCAAGTCGAACGGTAACAGGAAGAAGCTTGCTTCT
TTGCTGACGAGTGGCGGACGGGTGAGTAATGTCTGGGAAACTGCCTGATGGAGGGGGATA
ACTACTGGAAACGGTAGCTAATACCGCATAACGTCGCAAGACCAAAGAGGGGGTACCTTCG
GGCCTCTTGCCATCGGATGTGCCCAGATGGGATTAGCTAGTAGGTGGGGTAACGGCTCAC
CTAGGCGACGATCCCTAGCTGGTCTGAGAGGATGACCAGCCACACTGGAACTGAGACACG
GTCCAGACTCCTACGGGAGGCAGCAGTGGGGAATATTGCACAATGGGCGCAAGCCTGATG
CAGCCATGCCGCGTGTATGAAGAAGGCCTTCGGGTTGTAAAGTACTTTCAGCGGGGAGGA
AGGGAGTAAAGTTAATACCTTTGCTCATTGACGTTACCCGCAGAAGAAGCACCGGCTAAC
TCCGTGCCAGCAGCCGCGGTAATACGGAGGGTGCAAGCGTTAATCGGAATTACTGGGCGT
AAAGCGCACGCAGGCGGTTTGTTAAGTCAGATGTGAAATCCCCGGGCTCAACCTGGGAAC
TGCATCTGATACTGGCAAGCTTGAGTCTCGTAGAGGGGGGTAGAATTCCAGGTGTAGCGG
TGAAATGCGTAGAGATCTGGAGGAATACCGGTGGCGAAGGCGGCCCCCTGGACGAAGACT
GACGCTCAGGTGCGAAAGCGTGGGGAGCAAACAGGATTAGATACCCTGGTAGTCCACGCC
GTAAACGATGTCGACTTGGAGGTTGTGCCCTTGAGGCGTGGCTTCCGGAGCTAACGCGTT
AAGTCGACCGCCTGGGGAGTACGGCCGCAAGGTTAAAACTCAAATGAATTGACGGGGGCC
CGCACAAGCGGTGGAGCATGTGGTTTAATTCGATGCAACGCGAAGAACCTTACCTGGTCT
TGACATCCACGGAAGTTTTCAGAGATGAGAATGTGCCTTCGGGAACCGTGAGACAGGTGC
TGCATGGCTGTCGTCAGCTCGTGTTGTGAAATGTTGGGTTAAGTCCCGCAACGAGCGCAA
CCCTTATCCTTTGTTGCCAGCGGTCCGGCCGGGAACTCAAAGGAGACTGCCAGTGATAAA
CTGGAGGAAGGTGGGGATGACGTCAAGTCATCATGGCCCTTACGACCAGGGCTACACACG
TGCTACAATGGCGCATACAAAGAGAAGCGACCTCGCGAGAGCAAGCGGACCTCATAAAGT
GCGTCGTAGTCCGGATTGGAGTCTGCAACTCGACTCCATGAAGTCGGAATCGCTAGTAAT
CGTGGATCAGAATGCCACGGTGAATACGTTCCCGGGCCTTGTACACACCGCCCGTCACAC
CATGGGAGTGGGTTGCAAAAGAAGTAGGTAGCTTAACCTTCGGGAGGGCGCTTACCACTT
TGTGATTCATGACTGGGGTGAAGTCGTAACAAGGTAACCGTAGGGGAACCTGCGGTTGGA
TCACCTCCTTACCTTAAAGAAGCGTTCTTTGAAGTGCTCACACAGATTGTCTGATGAAAA
TGAGCAGTAAAACCTCTACAGGCTTGTAGCTCAGGTGGTTAGAGCGCACCCCTGATAAGG
GTGAGGTCGGTGGTTCAAGTCCACTCAGGCCTACCAAATTTGCACGGCAAATTTGAAGAG
GTTTTAACTACATGTTATGGGGCTATAGCTCAGCTGGGAGAGCGCCTGCTTTGCACGCAG

GAGGTCTGCGGTTCGATCCCGCATAGCTCCACCATCTCTGTAGTGATTAAATAAAAAATA
CTTCAGAGTGTACCTGCAAAGGTTCACTGCGAAGTTTTGCTCTTTAAAAATCTGGATCAA
GCTGAAAATTGAAACACTGAACAACGAAAGTTGTTCGTGAGTCTCTCAAATTTTCGCAAC
ACGATGATGAATCGAAAGAAACATCTTCGGGTTGTGAGGTTAAGCGACTAAGCGTACACG
GTGGATGCCCTGGCAGTCAGAGGCGATGAAGGACGTGCTAATCTGCGATAAGCGTCGGTA
AGGTGATATGAACCGTTATAACCGGCGATTTCCGAATGGGGAAACCCAGTGTGTTTCGAC
ACACTATCATTAACTGAATCCATAGGTTAATGAGGCGAACCGGGGGAACTGAAACATCTA
AGTACCCCGAGGAAAAGAAATCAACCGAGATTCCCCCAGTAGCGGCGAGCGAACGGGGAG
CAGCCCAGAGCCTGAATCAGTGTGTGTGTTAGTGGAAGCGTCTGGAAAGGCGTGCGATAC
AGGGTGACAGCCCCGTACACAAAAATGCACATGCTGTGAGCTCGATGAGTAGGGCGGGAC
ACGTGGTATCCTGTCTGAATATGGGGGGACCATCCTCCAAGGCTAAATACTCCTGACTGA
CCGATAGTGAACCAGTACCGTGAGGGAAAGGCGAAAAGAACCCCGGCGAGGGGAGTGAAA
AAGAACCTGAAACCGTGTACGTACAAGCAGTGGGAGCACGCTTAGGCGTGTGACTGCGTA
CCTTTTGTATAATGGGTCAGCGACTTATATTCTGTAGCAAGGTTAACCGAATAGGGGAGC
CGAAGGGAAACCGAGTCTTAACTGGGCGTTAAGTTGCAGGGTATAGACCCGAAACCCGGT
GATCTAGCCATGGGCAGGTTGAAGGTTGGGTAACACTAACTGGAGGACCGAACCGACTAA
TGTTGAAAAATTAGCGGATGACTTGTGGCTGGGGGTGAAAGGCCAATCAAACCGGGAGAT
AGCTGGTTCTCCCCGAAAGCTATTTAGGTAGCGCCTCGTGAATTCATCTCCGGGGGTAGA
GCACTGTTTCGGCAAGGGGGTCATCCCGACTTACCAACCCGATGCAAACTGCGAATACCG
GAGAATGTTATCACGGGAGACACACGGCGGGTGCTAACGTCCGTCGTGAAGAGGGAAACA
ACCCAGACCGCCAGCTAAGGTCCCAAAGTCATGGTTAAGTGGGAAACGATGTGGGAAGGC
CCAGACAGCCAGGATGTTGGCTTAGAAGCAGCCATCATTTAAAGAAAGCGTAATAGCTCA
CTGGTCGAGTCGGCCTGCGCGGAAGATGTAACGGGGCTAAACCATGCACCGAAGCTGCGG
CAGCGACACTATGTGTTGTTGGGTAGGGGAGCGTTCTGTAAGCCTGTGAAGGTGTGCTGT
GAGGCATGCTGGAGGTATCAGAAGTGCGAATGCTGACATAAGTAACGATAAAGCGGGTGA
AAAGCCCGCTCGCCGGAAGACCAAGGGTTCCTGTCCAACGTTAATCGGGGCAGGGTGAGT
CGACCCCTAAGGCGAGGCCGAAAGGCGTAGTCGATGGGAAACAGGTTAATATTCCTGTAC
TTGGTGTTACTGCGAAGGGGGGACGGAGAAGGCTATGTTGGCCGGGCGACGGTTGTCCCG
GTTTAAGCGTGTAGGCTGGTTTTCCAGGCAAATCCGGAAAATCAAGGCTGAGGCGTGATG
ACGAGGCACTACGGTGCTGAAGCAACAAATGCCCTGCTTCCAGGAAAAGCCTCTAAGCAT
CAGGTAACATCAAATCGTACCCCAAACCGACACAGGTGGTCAGGTAGAGAATACCAAGGC
GCTTGAGAGAACTCGGGTGAAGGAACTAGGCAAAATGGTGCCGTAACTTCGGGAGAAGGC
ACGCTGATATGTAGGTGAAGCGACTTGCTCGTGGAGCTGAAATCAGTCGAAGATACCAGC
TGGCTGCAACTGTTTATTAAAAACACAGCACTGTGCAAACACGAAAGTGGACGTATACGG
TGTGACGCCTGCCCGGTGCCGGAAGGTTAATTGATGGGGTTAGCCGCAAGGCGAAGCTCT
TGATCGAAGCCCCGGTAAACGGCGGCCGTAACTATAACGGTCCTAAGGTAGCGAAATTCC
TTGTCGGGTAAGTTCCGACCTGCACGAATGGCGTAATGATGGCCAGGCTGTCTCCACCCG
AGACTCAGTGAAATTGAACTCGCTGTGAAGATGCAGTGTACCCGCGGCAAGACGGAAAGA
CCCCGTGAACCTTTACTATAGCTTGACACTGAACATTGAGCCTTGATGTGTAGGATAGGT
GGGAGGCTTTGAAGTGTGGACGCCAGTCTGCATGGAGCCGACCTTGAAATACCACCCTTT
AATGTTTGATGTTCTAACGTTGACCCGTAATCCGGGTTGCGGACAGTGTCTGGTGGGTAG
TTTGACTGGGGCGGTCTCCTCCTAAAGAGTAACGGAGGAGCACGAAGGTTGGCTAATCCT
GGTCGGACATCAGGAGGTTAGTGCAATGGCATAAGCCAGCTTGACTGCGAGCGTGACGGC
GCGAGCAGGTGCGAAAGCAGGTCATAGTGATCCGGTGGTTCTGAATGGAAGGGCCATCGC
TCAACGGATAAAAGGTACTCCGGGGATAACAGGCTGATACCGCCCAAGAGTTCATATCGA
CGGCGGTGTTTGGCACCTCGATGTCGGCTCATCACATCCTGGGGCTGAAGTAGGTCCCAA
GGGTATGGCTGTTCGCCATTTAAAGTGGTACGCGAGCTGGGTTTAGAACGTCGTGAGACA
GTTCGGTCCCTATCTGCCGTGGGCGCTGGAGAACTGAGGGGGGCTGCTCCTAGTACGAGA

GGACCGGAGTGGACGCATCACTGGTGTTCGGGTTGTCATGCCAATGGCACTGCCCGGTAG
CTAAATGCGGAAGAGATAAGTGCTGAAAGCATCTAAGCACGAAACTTGCCCCGAGATGAG
TTCTCCCTGACTCCTTGAGAGTCCTGAAGGAACGTTGAAGACGACGACGTTGATAGGCCG
GGTGTGTAAGCGCAGCGATGCGTTGAGCTAACCGGTACTAATGAACCGTGAGGCTTAACC
TTACAACGCCGAAGGTGTTTTGGCGGATTGAGAGAAGATTTTCAGCCTGATACAGATTAA
ATCAGAACGCAGAAGCGGTCTGATAAAACAGAATTTGCCTGGCGGCAGTAGCGCGGTGGT
CCCACCTGACCCCATGCCGAACTCAGAAGTGAAACGCCGTAGCGCCGATGGTAGTGTGGG
GTCTCCTCATGCGAGAGTAGGGAACTGCCAGGCATCAAATAAAACGAAAGGCTCAGTCGG
AAGACTGGGCCTTTCGTTTTATCTGTTGTTTGTCGGTGAACGCTCTCCTGAGTAGGACAA
ATCCGCCGGGAGCGGATTTGAACGTTGCGAAGCAACGGCCCGGAGGGTGGCGGGCAGGAC
GCCCGCCATAAACTGCCAGGCATCAAATTAAGCAGAAGGCCATCCTGACGGATGGCCTTT
TTGCATTGGCGCAGAAAAAAATGCCGGATGCGACGCTGGCGCGTCTTAT
>R22
TGCAAAAAGGGCATCAAATGATGCCCTTTTAGTGCGCATTGCGTCAAATGTTATCGGCAA
TTAGCCCAGAACTTTAGCAACAACGCCCGCGCCAACGGTACGGCCGCCTTCACGGATTGC
GAAACGCAGACCGTCGTCCATCGCGATCGGGTGGATCAGGGTAACAACCATTTTGATGTT
GTCGCCCGGCATTACCATCTCTACGCCTTCCGGCAGTTCGATGGTACCAGTCACGTCAGT
AGTACGGAAGTAGAACTGCGGACGGTAGCCTTTGAAGAACGGAGTATGACGGCCGCCTTC
ATCTTTGGACAGAATGTACACTTCAGATTCGAACTTGGTGTGCGGCTTGATGGTGCCCGG
CTTAGCCAGTACCTGACCACGTTCGATTTCTTCACGTTTGATACCACGCAGCAGAACACC
TACGTTCTCACCAGCACGGCCTTCGTCCAGCAGTTTGCGGAACATTTCAACGCCAGTACA
GGTAGACTTCTGAGTCTCTTTGATACCAACGATTTCAACTTCTTCACCAACTTTGATGAT
ACCGCGTTCTACACGACCGGTAACAACGGTACCACGACCGGAGATGGAGAATACGTCTTC
GATCGGCAGCAGGAACGGCTTGTCAATCGCACGCTCTGGTTCCGGAATATAAGAATCCAG
GAAGCCAGCCAGTTCCAGGATTTTCGCTTCCCACTCTGCGTCGCCTTCCAGCGCTTTCAG
AGCAGAACCACGAACGATCGGAGTGTCGTCGCCCGGGAAGTCGTACTGAGACAGAAGTTC
ACGAACTTCCATTTCAACCAGTTCCAGCAGCTCTTCGTCATCAACCATGTCGCATTTGTT
CAGGAACACGATGATGTACGGAACGCCTACCTGACGACCCAGCAGGATGTGCTCACGAGT
CTGCGGCATCGGGCCGTCAGTCGCAGCAACTACCAGGATCGCGCCGTCCATCTGAGCAGC
ACCGGTGATCATGTTTTTAACATAGTCGGCGTGCCCCGGGCAGTCTACGTGTGCGTAGTG
ACGGGTCGGGGTGTCGTATTCAACGTGAGAAGTGTTGATGGTGATACCACGAGCTTTTTC
TTCCGGCGCGTTATCGATCTGGTCGAATGCACGAGCAGCACCGCCGTAGGTTTTAGCCAG
TACGGTAGTGATTGCAGCGGTCAGAGTAGTTTTACCATGGTCAACGTGGCCGATAGTACC
AACGTTAACGTGCGGTTTTGTACGTTCAAACTTTTCTTTAGACA
>R23
TCCCTATAATGCGCCTCCATCGACACGGCGGATGTGAATCACTTCACACAAACAGCCGGT
TCGGTTGAAGAGAAAAATCCTGAAATTCAGGGTTGACTCTGAAAGAGGAAAGCGTAATAT
ACGCCACCTCGCGACAGTGCGCTAAAGCGCGTCGCAACTGCTCTTTAACAATTTATCAGA
CAATCTGTGTGGGCACTCGAAGATACGGATTCTTAACGTCGCAAGACGAAAAATGAATAC
CAAGTCTCAAGAGTGAACACGTAATTCATTACGAAGTTTAATTCTTTGAGCATCAAACTT
TTAAATTGAAGAGTTTGATCATGGCTCAGATTGAACGCTGGCGGCAGGCCTAACACATGC
AAGTCGAACGGTAACAGGAAGAAGCTTGCTTCTTTGCTGACGAGTGGCGGACGGGTGAGT
AATGTCTGGGAAACTGCCTGATGGAGGGGGATAACTACTGGAAACGGTAGCTAATACCGC
ATAACGTCGCAAGACCAAAGAGGGGGACCTTCGGGCCTCTTGCCATCGGATGTGCCCAGA
TGGGATTAGCTAGTAGGTGGGGTAACGGCTCACCTAGGCGACGATCCCTAGCTGGTCTGA
GAGGATGACCAGCCACACTGGAACTGAGACACGGTCCAGACTCCTACGGGAGGCAGCAGT
GGGGAATATTGCACAATGGGCGCAAGCCTGATGCAGCCATGCCGCGTGTATGAAGAAGGC
CTTCGGGTTGTAAAGTACTTTCAGCGGGGAGGAAGGGAGTAAAGTTAATACCTTTGCTCA

TTGACGTTACCCGCAGAAGAAGCACCGGCTAACTCCGTGCCAGCAGCCGCGGTAATACGG
AGGGTGCAAGCGTTAATCGGAATTACTGGGCGTAAAGCGCACGCAGGCGGTTTGTTAAGT
CAGATGTGAAATCCCCGGGCTCAACCTGGGAACTGCATCTGATACTGGCAAGCTTGAGTC
TCGTAGAGGGGGGTAGAATTCCAGGTGTAGCGGTGAAATGCGTAGAGATCTGGAGGAATA
CCGGTGGCGAAGGCGGCCCCCTGGACGAAGACTGACGCTCAGGTGCGAAAGCGTGGGGAG
CAAACAGGATTAGATACCCTGGTAGTCCACGCCGTAAACGATGTCGACTTGGAGGTTGTG
CCCTTGAGGCGTGGCTTCCGGAGCTAACGCGTTAAGTCGACCGCCTGGGGAGTACGGCCG
CAAGGTTAAAACTCAAATGAATTGACGGGGGCCCGCACAAGCGGTGGAGCATGTGGTTTA
ATTCGATGCAACGCGAAGAACCTTACCTGGTCTTGACATCCACGGAAGTTTTCAGAGATG
AGAATGTGCCTTCGGGAACCGTGAGACAGGTGCTGCATGGCTGTCGTCAGCTCGTGTTGT
GAAATGTTGGGTTAAGTCCCGCAACGAGCGCAACCCTTATCCTTTGTTGCCAGCGGTCCG
GCCGGGAACTCAAAGGAGACTGCCAGTGATAAACTGGAGGAAGGTGGGGATGACGTCAAG
TCATCATGGCCCTTACGACCAGGGCTACACACGTGCTACAATGGCGCATACAAAGAGAAG
CGACCTCGCGAGAGCAAGCGGACCTCATAAAGTGCGTCGTAGTCCGGATTGGAGTCTGCA
ACTCGACTCCATGAAGTCGGAATCGCTAGTAATCGTGGATCAGAATGCCACGGTGAATAC
GTTCCCGGGCCTTGTACACACCGCCCGTCACACCATGGGAGTGGGTTGCAAAAGAAGTAG
GTAGCTTAACCTTCGGGAGGGCGCTTACCACTTTGTGATTCATGACTGGGGTGAAGTCGT
AACAAGGTAACCGTAGGGGAACCTGCGGTTGGATCACCTCCTTACCTTAAAGAAGCGTTC
TTTGCAGTGCTCACACAGATTGTCTGATAGAAAGTGAAAAGCAAGGCGTCTTGCGAAGCA
GACTGATACGTCCCCTTCGTCTAGAGGCCCAGGACACCGCCCTTTCACGGCGGTAACAGG
GGTTCGAATCCCCTAGGGGACGCCACTTGCTGGTTTGTGAGTGAAAGTCACCTGCCTTAA
TATCTCAAAACTCATCTTCGGGTGATGTTTGAGATATTTGCTCTTTAAAAATCTGGATCA
AGCTGAAAATTGAAACACTGAACAACGAAAGTTGTTCGTGAGTCTCTCAAATTTTCGCAA
CACGATGATGAATCGTAAGAAACATCTTCGGGTTGTGAGGTTAAGCGACTAAGCGTACAC
GGTGGATGCCCTGGCAGTCAGAGGCGATGAAGGACGTGCTAATCTGCGATAAGCGTCGGT
AAGGTGATATGAACCGTTATAACCGGCGATTTCCGAATGGGGAAACCCAGTGTGTTTCGA
CACACTATCATTAACTGAATCCATAGGTTAATGAGGCGAACCGGGGGAACTGAAACATCT
AAGTACCCCGAGGAAAAGAAATCAACCGAGATTCCCCCAGTAGCGGCGAGCGAACGGGGA
GCAGCCCAGAGCCTGAATCAGTGTGTGTGTTAGTGGAAGCGTCTGGAAAGGCGCGCGATA
CAGGGTGACAGCCCCGTACACAAAAATGCACATGCTGTGAGCTCGATGAGTAGGGCGGGA
CACGTGGTATCCTGTCTGAATATGGGGGGACCATCCTCCAAGGCTAAATACTCCTGACTG
ACCGATAGTGAACCAGTACCGTGAGGGAAAGGCGAAAAGAACCCCGGCGAGGGGAGTGAA
AAAGAACCTGAAACCGTGTACGTACAAGCAGTGGGAGCACGCTTAGGCGTGTGACTGCGT
ACCTTTTGTATAATGGGTCAGCGACTTATATTCTGTAGCAAGGTTAACCGAATAGGGGAG
CCGAAGGGAAACCGAGTCTTAACTGGGCGTTAAGTTGCAGGGTATAGACCCGAAACCCGG
TGATCTAGCCATGGGCAGGTTGAAGGTTGGGTAACACTAACTGGAGGACCGAACCGACTA
ATGTTGAAAAATTAGCGGATGACTTGTGGCTGGGGGTGAAAGGCCAATCAAACCGGGAGA
TAGCTGGTTCTCCCCGAAAGCTATTTAGGTAGCGCCTCGTGAATTCATCTCCGGGGGTAG
AGCACTGTTTCGGCAAGGGGGTCATCCCGACTTACCAACCCGATGCAAACTGCGAATACC
GGAGAATGTTATCACGGGAGACACACGGCGGGTGCTAACGTCCGTCGTGAAGAGGGAAAC
AACCCAGACCGCCAGCTAAGGTCCCAAAGTCATGGTTAAGTGGGAAACGATGTGGGAAGG
CCCAGACAGCCAGGATGTTGGCTTAGAAGCAGCCATCATTTAAAGAAAGCGTAATAGCTC
ACTGGTCGAGTCGGCCTGCGCGGAAGATGTAACGGGGCTAAACCATGCACCGAAGCTGCG
GCAGCGACGCTTATGCGTTGTTGGGTAGGGGAGCGTTCTGTAAGCCTGCGAAGGTGTGCT
GTGAGGCATGCTGGAGGTATCAGAAGTGCGAATGCTGACATAAGTAACGATAAAGCGGGT
GAAAAGCCCGCTCGCCGGAAGACCAAGGGTTCCTGTCCAACGTTAATCGGGGCAGGGTGA
GTCGACCCCTAAGGCGAGGCCGAAAGGCGTAGTCGATGGGAAACAGGTTAATATTCCTGT
ACTTGGTGTTACTGCGAAGGGGGGACGGAGAAGGCTATGTTGGCCGGGCGACGGTTGTCC

CGGTTTAAGCGTGTAGGCTGGTTTTCCAGGCAAATCCGGAAAATCAAGGCTGAGGCGTGA
TGACGAGGCACTACGGTGCTGAAGCAACAAATGCCCTGCTTCCAGGAAAAGCCTCTAAGC
ATCAGGTAACATCAAATCGTACCCCAAACCGACACAGGTGGTCAGGTAGAGAATACCAAG
GCGCTTGAGAGAACTCGGGTGAAGGAACTAGGCAAAATGGTGCCGTAACTTCGGGAGAAG
GCACGCTGATATGTAGGTGAGGTCCCTCGCGGATGGAGCTGAAATCAGTCGAAGATACCA
GCTGGCTGCAACTGTTTATTAAAAACACAGCACTGTGCAAACACGAAAGTGGACGTATAC
GGTGTGACGCCTGCCCGGTGCCGGAAGGTTAATTGATGGGGTTAGCGCAAGCGAAGCTCT
TGATCGAAGCCCCGGTAAACGGCGGCCGTAACTATAACGGTCCTAAGGTAGCGAAATTCC
TTGTCGGGTAAGTTCCGACCTGCACGAATGGCGTAATGATGGCCAGGCTGTCTCCACCCG
AGACTCAGTGAAATTGAACTCGCTGTGAAGATGCAGTGTACCCGCGGCAAGACGGAAAGA
CCCCGTGAACCTTTACTATAGCTTGACACTGAACATTGAGCCTTGATGTGTAGGATAGGT
GGGAGGCTTTGAAGTGTGGACGCCAGTCTGCATGGAGCCGACCTTGAAATACCACCCTTT
AATGTTTGATGTTCTAACGTTGACCCGTAATCCGGGTTGCGGACAGTGTCTGGTGGGTAG
TTTGACTGGGGCGGTCTCCTCCTAAAGAGTAACGGAGGAGCACGAAGGTTGGCTAATCCT
GGTCGGACATCAGGAGGTTAGTGCAATGGCATAAGCCAGCTTGACTGCGAGCGTGACGGC
GCGAGCAGGTGCGAAAGCAGGTCATAGTGATCCGGTGGTTCTGAATGGAAGGGCCATCGC
TCAACGGATAAAAGGTACTCCGGGGATAACAGGCTGATACCGCCCAAGAGTTCATATCGA
CGGCGGTGTTTGGCACCTCGATGTCGGCTCATCACATCCTGGGGCTGAAGTAGGTCCCAA
GGGTATGGCTGTTCGCCATTTAAAGTGGTACGCGAGCTGGGTTTAGAACGTCGTGAGACA
GTTCGGTCCCTATCTGCCGTGGGCGCTGGAGAACTGAGGGGGGCTGCTCCTAGTACGAGA
GGACCGGAGTGGACGCATCACTGGTGTTCGGGTTGTCATGCCAATGGCACTGCCCGGTAG
CTAAATGCGGAAGAGATAAGTGCTGAAAGCATCTAAGCACGAAACTTGCCCCGAGATGAG
TTCTCCCTGACTCCTTGAGAGTCCTGAAGGAACGTTGAAGACGACGACGTTGATAGGCCG
GGTGTGTAAGCGCAGCGATGCGTTGAGCTAACCGGTACTAATGAACCGTGAGGCTTAACC
TTACAACGCCGAAGGTGTTTTGGCGGATTGAGAGAAGATTTTCAGCCTGATACAGATTAA
ATCAGAACGCAGAAGCGGTCTGATAAAACAGAATTTGCCTGGCGGCAGTAGCGCGGTGGT
CCCACCTGACCCCATGCCGAACTCAGAAGTGAAACGCCGTAGCGCCGATGGTAGTGTGGG
GTCTCCCCATGCGAGAGTAGGGAACTGCCAGGCATCAAATTA
